# Properties and Phylogeny of 76 Families of Bacterial and Eukaryotic Organellar Outer Membrane Pore-Forming Proteins

**DOI:** 10.1371/journal.pone.0152733

**Published:** 2016-04-11

**Authors:** Bhaskara L. Reddy, Milton H. Saier

**Affiliations:** Department of Molecular Biology, Division of Biological Sciences, University of California at San Diego, La Jolla, California, United States of America; University of Roskilde, DENMARK

## Abstract

We here report statistical analyses of 76 families of integral outer membrane pore-forming proteins (OMPPs) found in bacteria and eukaryotic organelles. 47 of these families fall into one superfamily (SFI) which segregate into fifteen phylogenetic clusters. Families with members of the same protein size, topology and substrate specificities often cluster together. Virtually all OMPP families include only proteins that form transmembrane pores. Nine such families, all of which cluster together in the SFI phylogenetic tree, contain both α- and β-structures, are multi domain, multi subunit systems, and transport macromolecules. Most other SFI OMPPs transport small molecules. SFII and SFV homologues derive from Actinobacteria while SFIII and SFIV proteins derive from chloroplasts. Three families of actinobacterial OMPPs and two families of eukaryotic OMPPs apparently consist primarily of α-helices (α-TMSs). Of the 71 families of (putative) β-barrel OMPPs, only twenty could not be assigned to a superfamily, and these derived primarily from Actinobacteria (1), chloroplasts (1), spirochaetes (8), and proteobacteria (10). Proteins were identified in which two or three full length OMPPs are fused together. Family characteristic are described and evidence agrees with a previous proposal suggesting that many arose by adjacent β-hairpin structural unit duplications.

## Introduction

Most bacteria, including all Gram-negative bacteria and some Gram-positive Firmicutes and Actinobacteria, as well as mitochondria and chloroplasts of eukaryotes, have envelopes consisting of two membranes, an inner cytoplasmic or matrix membrane and an outer membrane with special protective functions [1]. In Gram-negative bacteria and eukaryotic organelles, most integral outer membrane pore-forming proteins (OMPPs) contrast with integral inner membrane proteins with respect to their structural features. While integral inner membrane proteins generally possess transmembrane α-helical segments (α-TMSs), integral outer membrane proteins (OMPs) usually consist of transmembrane β-strands (β-TMSs) that form β-barrels [2]. Large proportions of these β-barrel proteins are OMPPs that non-selectively allow passage of molecules across the outer permeability barrier. These proteins also serve as cell surface antigens that provide targets for vaccine development [3, 4]. However, many other outer membrane pore-forming proteins exhibit substrate selectivity, and we here designate porins and all other outer membrane pore-forming proteins collectively as OMPPs [5].

Bioinformatic analyses and evolutionary considerations have led to the conclusion that many proteins have arisen from ancient peptide modules coded for by genes that underwent repeated intragenic multiplication (duplication, triplication, quadruplication, etc.) to generate larger proteins [6–8]. Replication slippage provides one mechanism for the generation of multiple repeats, and stable protein complexes have apparently evolved more frequently from identical units than from dissimilar ones [9]. In fact, some of the most popular folds found in proteins include structural repeats [8]. It has been argued that these repeat sequences arose by divergent rather than convergent evolutionary processes, a conclusion that in many cases, has been extensively documented [6, 7].

Over the past two decades, our laboratory has studied the evolution of numerous integral membrane transport proteins consisting largely of α-TMSs [6, 7, 10–16] (see the Transporter Classification Database, TCDB; www.tcdb.org) [17–20]. Different families have evolved via different routes, most frequently beginning with small units including one, two, three or four α-TMSs, which appear in current transporters as repeat units [6, 7, 10, 11]. In fact, distinct pathways have been documented, allowing one to conclude that several of these families have evolved from their precursors independently of each other [21]. Examples include the OPT family (TC#2.A.67) of peptide transporters which evolved from a 2 α-TMS hairpin repeat unit via the pathway: 2 → 4 → 8 → 16 TMSs [10]. Two other families (Mitochondrial Carriers, MC; TC# 2.A.29 [22], and ABC1; 3.A.1 [23]) evolved via triplication of a two α-TMS hairpin structure to give domains of six TMSs. Several families, including the MIP [24], LysE [25], [26], MFS [27] and ABC2 [23, 28] families, probably evolved initially via duplication of a three α-TMS unit, and other families evolved by duplication of a four α-TMS segment (i.e., the ABC3 [23, 28] and TOG [29] superfamilies). Although the possibility that some of these repeat units arose from smaller units, as has been proposed [23], we have suggested that the α-type transport proteins have evolved multiple times independently to satisfy the needs of the cell to mediate uptake and export of a variety of molecules [6, 7].

Our laboratory has identified over 1000 families of transport proteins and over 60 superfamilies of integral membrane transport proteins (see superfamily hyperlink in TCDB) [6, 7, 18, 20]. These superfamilies include six types of α-helical channel proteins, four pore-forming toxin types, seven holin types, one viral envelope type, five toxic channel-forming peptide types, eleven secondary carrier types, six primary active transporter types, and two group translocator types. In any one of these superfamilies, the protein members always exhibit the same internal repeat units. However, some superfamilies have diversified to include channels, carriers and primary carriers [26, 30].

In 2010, Remmert et al. [31] proposed that most outer membrane β-barrel proteins have a common origin, being derived from a single ancestral ββ-hairpin structure. This suggestion was based on three types of experimental evidence. First, the authors used transitive profile searching (a search with BLAST is performed, and all significantly matched sequences are used in new searches [32]; second, they identified repeat signature sequences in some OMPPs in which the repeated sequence units coincided with the proposed ββ-hairpin repeats, and third, they provided evidence that similarity between some of the outer membrane β-barrel hairpins could not be explained by structural or membrane constraints on their sequences. This last consideration addressed the issue of convergent versus divergent evolution, responsible for the sequence similarity observed. They rejected the notion of convergent pathways in favor of divergent pathways, suggesting that the proteins arose by amplification and recombination of ββ-hairpin modules that might previously have evolved as RNA co-factors [31]. The Protein Family (Pfam) Database also provides evidence, that many β-barrel OMPP families are related by common ancestry [33, 34].

As noted above, our laboratory has focused primarily on superfamilies of α-TMS transport proteins with little emphasis on outer membrane pore-forming proteins (OMPPs) [6, 7, 10–12, 21, 35]. In order to define superfamily relationships, we have developed statistical means to evaluate the probability of homology, e.g., common origin [36]. Our standard methods involve the use of computer programs that use the Superfamily Principle to determine the significance of sequence similarities [34]. The Superfamily Principle states that if protein A is homologous to protein B, and protein B is homologous to protein C, then protein A must be homologous to protein C, regardless of the degree of sequence similarity observed between these two proteins. It should be noted that homology is an absolute term meaning, “derived from a common ancestor” and does not imply a specific degree of sequence similarity. Thus, two proteins or protein domains are homologous if they share common descent.

We have decided to use the methods developed in our laboratory [36] to independently examine the possibility of common origin of recognized OMPP families in TCDB using rigorous quantitative statistical approaches. We use these methods to establish the relationships of the various families to each other and describe their families’ characteristics, based on our bioinformatic analyses as well as the published literature [37]. To our surprise, and in contrast to previous analyses with α-helical type transport proteins [6, 7, 12], we observed a remarkable degree of sequence similarity among many of the 76 currently recognized families of OMPPs and putative OMPPs included in TCDB as of 5/2015 (see Table 1).

**Table 1:** Characteristics of 76 OMPP families in TCDB.

TCDB #	Family name & abbreviation	Organismal types^a^	Average protein size^b^	Family Size^c^	Super family assign-ment	# β- TMSs^d^; Established or predicted	CDD/Pfam Superfamily^e^
1.B.1	General Bacterial OMPP (GBP)	Proteobacteria; Acidobacteria; Chlorobi	347±51	3407	I	16*	Gram neg OMPPs OM channels
1.B.2	Chlamydial OMPP (CP)	Chlamydiae	328±60	1018	I	16	Chlam–OMP
1.B.3	Sugar OMPP (SP)	Proteobacteria; Verrucomicrobia; Nitrospirae; Planctomycetes; Aquificae	453±48	1833	I	18*	MaltoOMPP-like OM Channels
1.B.4	Brucella- Rhizobium OMPP (BRP)	Proteobacteria	266±99	1165	I	12*	Gcw chp; COG3637 OMP b-brl; DUF4104
1.B.5	Pseudomonas OprP OMPP (POP)	Proteobacteria; Planctomycetes; Bacteroidetes; Verrucomicrobia; Aquificae	429±58	692	I	16*	OMPP O P
1.B.6	OmpA-OmpF OMPP (OOP)	Proteobacteria; Bacteroidetes; Fusobacteria; Firmicutes; Spirochaetes; Verrucomicrobia; Chlorobi	264±85	3804	I	8*	OmpA membrane Omp b-brl; OmpA C-like; PRK10808
1.B.7	Rhodobacter PorCa OMPP (RPP)	Proteobacteria	333±32	142	I	16*	OMPP 4 OM Channels
1.B.8	Mitochondrial and Plastid OMPP (MPP)	Fungi; Metazoa; Viridiplantae; Euglenozoa; Alveolata; Amoebozoa; Stramenopiles	290±14	962	I	19*	OMPP3 VDAC
1.B.9	FadL Outer Membrane OMPP ((FadL)	Proteobacteria; Bacteroidetes; Nitrospirae; Spirochaetae; Chlamydiae	434±34	2969	I	14*	PRK10716 Toluene X
1.B.10	Nucleoside-specific Channel-forming Outer Membrane OMPP (Tsx)	Proteobacteria	278±13	756	I	12*	PRK15106 Channel Tsx
1.B.11	Outer Membrane Fimbrial Usher OMPP (FUP)	Proteobacteria; Spirochaetae; Cyanobacteria; *Deinococcus/ Thermus*	830±32	5000	I	24* (α- & β- structure)	PapC N; Usher; PRK15213; PapC C
1.B.12	Autotransporter-1 (AT-1)	Proteobacteria	1345± 575	4679	I	12* (α- & β- structure)	PRK09945; Autotrans barl; PL Passenger AT
1.B.13	Alginate Export OMPP (AEP)	Proteobacteria; Aquificae; Spirochaetae; Acidobacteria; Verrucomicrobia	507±96	206	I	18*	DUF4104 SPOR
1.B.14	Outer Membrane Receptor (OMR)	Proteobacteria; Bacteroidetes; Cyanobacteria; Spirochaetae; Chloroflexi; Chlamydiae	782± 135	5000	I	22*	Ligand gated Channel OM channels
1.B.15	Raffinose OMPP (RafY)	Proteobacteria	377±63	318	I	16	SugarOMPP
1.B.16	Short Chain Amide and Urea OMPP ((SAP)	Proteobacteria; Aquificae; Thermodesulfo-bacteria; Bacteroidetes; Nitrospirae; Planctomycetes; Acidobacteria; Chrysiogenetes; Gemmatimnadetes;Synergistetes sp	414±32	135	I	16	OMPP O P
1.B.17	Outer Membrane Factor (OMF)	Proteobacteria; Chlamydiae; Firmicutes	472±30	5000	I	12* (α- & β- structure)	TolC
1.B.18	Outer Membrane Auxillary (OMA) Protein	Proteobacteria; Bacteroidetes; Chlamydiae	393±82	3180	I	1α-helix (α- & β- structure)	Poly export; SLBB
1.B.19	Glucose- selective OprB OMPP (OprB)	Proteobacteria; Cyanobacteria; Planctomycetes; Acidobacteria	459±36	1550	I	16*	OprB
1.B.20	Two- Partner Secretion (TPS)	Proteobacteria; Chlorobi	589±65	2651	I	16* (α- & β- structure)	POTRA 2; ShiB; FhaC
1.B.21	OmpG OMPP (OmpG)	Proteobacteria; Fusobacteria	316±39	169	I	14*	OMPP OmpG
1.B.22	Bacterial Outer Membrane Secretin (Secretin)	Proteobacteria; Chlamydiae	555± 151	5000	I	12 (12 sub units) (α- & β- structure)	Secretin N; Secretin type II gspD
1.B.23	Cyanobacterial OMPP (CBP)	Cyanobacteria; Proteobacteria; Chlamydiae; Acidobacteria; Nitrospirae; Verrucomicrobia; Synergistes; Fibrobacteres; Firmicutes	457±56	1862	I	16	OprB; SLH
1.B.24	Mycobacterial OMPP (MBP)	Actinobacteria; Proteobacteria	275±84	209	II	16*	MspA
1.B.25	Outer Membrane OMPP (Opr)	Proteobacteria; Aquificae; Lentisphaerae	428±44	3827	I	18*	OprD
1.B.26	Cyclodextrin OMPP (CDP)	Proteobacteria	353±27	30	I	16	OMPP 2
1.B.27	*Helicobacter* Outer Membrane OMPP (HOP)	Proteobacteria	488±182	4926		10	HP OMP
1.B.28	Plastid Outer Envelop OMPP of 24kDa (OEP24)	Viridiplantae	234±35	66	III	12	
1.B.29	Plastid Outer Envelop OMPP of 21kDa (OEP21)	Viridiplantae	208±54	53		8	
1.B.30	Plastid Outer Envelop OMPP of 16kDa (OEP16)^f^	Viridiplantae; Stramenopiles	163±26	205	IV	4 α- helices	Tim17
1.B.31	*Camphylobacter jejuni* Major Outer Membrane OMPP (MomP)	Proteobacteria	418±41	365	I	16	Campylo MoMP
1.B.32	Fusobacterial Outer Membrane OMPP (FomA)	Fusobacteria; Proteobacteria	331±32	77	I	14	
1.B.33	Outer Membrane Protein Insertion OMPP (OmpIP)	Proteobacteria; Planctomycetes; Viridiplantae; Nitrospirae; Fungi; Spirochaetes	594±229	5000	I	16* (α- & β- structure)	Bac Surface Ag
1.B.34	Corynebacterial OMPP A (PorA)	Actinobacteria	44±2	8	V	1 α-helix	
1.B.35	Oligogalacturonate-specific OMPP (KdgM)	Proteobacteria	249±21	527	I	12*	KdgM
1.B.36	*Borrelia* OMPP p13 (BP-p13)	Spirochaetes	169±13	102		12	Borrelia P13
1.B.37	Leptospira OMPP OmpL1 (LP-OmpL1)	Spirochaetes	335±21	149		12	OMPP Ompl1
1.B.38	Treponema OMPP Major Surface Protein (TP-MSP)	Spirochaetes	532±54	363		24	MOSP N; MOSP C
1.B.39	Bacterial OMPP, OmpW (OmpW)	Proteobacteria	227±10	2173	I	8*	PRK10959 OmpW
1.B.40	Autotransporter-2 (AT-2)	Proteobacteria Firmicutes Fusobacteria; Mollicutes; Chlamydiae	1270± 1290	4079		12*	LbR like; YadA- Ancher
1.B.41	Corynebacterial OMPP B (PorB)	Actinobacteria	139±11	23		4 α-helices	PorB
1.B.42	Outer Membrane Lipopolysaccharide Export OMPP (LPS-EP)	Proteobacteria; Aquificae; Chlorobi; Bacteroidetes; Verrucomicrobia; Spirochaetes	750± 223	2933	I	26* (α- & β- structure)	OstA C
1.B.43	*Coxiella* OMPP P1 (CPP1)	Proteobacteria	242±45	33	I	8	OMP b-brl
1.B.44	Probable Protein Translocating *Porphyromonas gingivalis* OMPP (PorT)	Bacteroidetes	235±26	248	I	8	OMP b-brl 2
1.B.45	*Treponema* OMPP (T-Por)	Spirochaetes	297±19	32		12*	
1.B.46	Outer Membrane LolAB Lipoprotein Insertion Apparatus (LolAB)	Proteobacteria	208±5	1488		12*	LolA
1.B.47	Plastid Outer Envelope OMPP of 37 kDa (OEP37)	Viridiplantae	334±8	73	III	14	Arena RNA pol
1.B.48	Curli Fiber Subunit, CsgA, OMPP, CsgG (GsgG)	Proteobacteria; Chlorobi; Cyanobacteria; Firmicutes; Bacterioidetes; Spirochaetes; Thermatogae; Thermus; Aquificae	283±48	502		10	TolB N; CsgE; CsgF; Surface Ag 2
1.B.49	*Anaplasma* P44 (A-P44) OMPP	Proteobacteria	266±30	4199	I	8	Surface Ag 2
1.B.50	Acid Fast Bacterial, Outer Membrane OMPP (AFB-OMP)	Actinobacteria; Armatimonadetes	306±12	395		2	DUF3186
1.B.51	Oms66 OMPP (Oms66P)	Spirochaetes	317±267	142		26	Attachment P66
1.B.52	Oms28 OMPP (Oms28P)	Spirochaetes; Proteobacteria	273±23	51		2	OMS 28 OMPP
1.B.53	Filamentous Phage g3p Channel-forming Protein (FP-g3p)	Proteobacteria	429±4	237		13*	Phage Coat A
1.B.54	Intimin/Invasin (Int/Inv) or Autotransporter-3 (AT-3)	Proteobacteria; Chlamydiae; Cyanobacteria; Chlorobi	793±572	2881	I	12*	DUF3442; Big 1; BID 1; Big 2
1.B.55	Poly Acetyl Glucosamine OMPP (PgaA)^g^	Proteobacteria; Planctomycetes	786±155	719	I	28 (α- & β- structure)	PgaA
1.B.56	Spirochaete Outer Membrane OMPP (S-OMP)	Spirochaetes	322±14	64		12	
1.B.57	*Legionella* Major-Outer Membrane Protein (LM-OMP)	Proteobacteria; Planctomycetes; Elusimicrobia; Bacteroidetes	370±52	125	I	12	Legionella OMP
1.B.58	Nocardial Hetero-oligomeric Cell Wall Channel (NfpA/B)	Actinobacteria	235±24	282	II	8	MSPA
1.B.59	Outer Membrane OMPP (PorH)	Actinobacteria	61±5	13	V	1 α-helix	
1.B.60	Omp50 OMPP (Omp50 OMPP)	Proteobacteria; Deferribacteres	540±49	201	I	18	DUF373
1.B.61	Delta-Proteobacterial OMPP (Delta-OMPP)	Proteobacteria	456±13	44	I	18	OM Channels
1.B.62	Putative Bacterial OMPP (PBP)	Proteobacteria; Chlorobi; Verrucomicrobia; Lentisphaerae	505±71	258	I	18	DUF3373; DUF1097
1.B.63	Imipenum resistance-associated OMPP (CarO)	Proteobacteria; Cyanobacteria; Acidobacteria; Bacteroidetes	297±74	274		12*	PRK13856
1.B.64	Brucella Omp2 OMPP (B-Omp2) Family	Proteobacteria	407±30	37		2	Brucella OMP2 Superfamily
1.B.65	The Outer Membrane OMPP OpcA (OpcA) Family	Proteobacteria; Elusimicrobia; Chlorobi	254±46	84		13*	OpcA Superfamily
1.B.66	Putative Beta-Barrel OMPP-2 (BBP2)	Proteobacteria; Bacteroidetes; Planctomycetes; Spirochaetes; Verrucomicrobia; Lentisphaerae	419±50	298	I	10	DUF1597
1.B.67	Putative Beta Barrel OMPP-4 (BBP4)	Proteobacteria; Thermodesulfo-bacteria; Ignavibacteria; Aquificae	400±45	81	I	16	OM Channels
1.B.68	Putative Beta Barrel OMPP-5 (BBP5)	Proteobacteria	188±7	397	I	8	OMP w GlyGly
1.B.69	Peroxisomal Membrane OMPP4 (PxMP4)^f^	Protozoa; Plants; Fungi; Animals; Alveolata; Euglenozoa	220±14	294	IV	4 α-helices	Tim17
1.B.70	Outer Membrane Channels (OMC)	Proteobacteria; Chlorobi; Planctomycetes	468±59	495	I	16	OM Channels; OMPP 2
1.B.71	Proteobacterial/ Verrumicrobial OMPP (PVP)	Proteobacteria; Verrumicrobia;	247±18	37	I	12	Gcw-chp
1.B.72	Protochlamydial Outer Membrane OMPP (PomS/T)	Chlamydiae; Proteobacteria; Bacteroidetes; Nitrospirae	444± 243	10	I	12	Autotransporter; DUF1551; Omptin
1.B.73	Capsule Biogenesis/Assembly (CBA)	Proteobacteria; Thermodesulfo bacteria; Bacteroidetes	514 ± 50	304	I	18*	Caps assemb Wzi
1.B.74	Outer Membrane Beta Barrel L32 Protein (OmpL32)	Spirochaetes	275±26	50		10	
1.B.75	DUF481 Putative Beta Barrel OMPP (DUF481)	Chlamydiae; Proteobacteria; Bacteroidetes; Nitrospirae	277±41	506		12	DUF481
1.B.76	Copper Resistance Putative OMPP (CopB)	Proteobacteria; Thermodesulfo bacteria; Bacteroidetes	424 ± 265	537		10	CopB
1.B.77 ^h^	Chloroplast Outer Envelope OMPP (OEP23)	Plants; Actinobacteria; Stramenopiles; Deinococcus/ Thermus	232±49	503		9	DUF 1990
1.B.78 ^h^	DUF3374 Electron Transport-associated OMPP (ETOMPP)	Proteobacteria	654± 242	345	I	26	UM channels; DUF 3374
1.B.79 ^h^	OMPP-Sphingomyelinase Fusion Protein (SpmT)	Actinobacteria	450± 124	550		8	EEP

^a^ Phyla are listed in approximate order of representation in each family.

^b^ Values are presented in numbers of amino acyl residues (aas) per polypeptide chain ± standard deviations.

^c^ Family size is expressed in terms of numbers of homologues retrieved when TC protein 1.B.X.1.1 was PSI-BLASTed against the NCBI NR Protein Database on 10/14.

^d^ Number of established (indicated by an asterisk) or predicted numbers of transmembrane β-strands (β-TMSs). Three families, 1.B.34, 1.B.41, and 1.B.59, are established OMPPs of Actinobacteria, but their transmembrane segments are α-helical, and two of these families, 1.B.34 and 1.B.59, possess a single transmembrane α-helix and appear to be related (T. Su and M.H. Saier, unpublished observation).

^e^ CDD (Conserved Domain Database); a blank indicates a family not recognized by CDD.

^f^ Members of the OEP16 family (1.B.30) and the PxMP4 family (1.B.69) form a single superfamily together with Tim17, Tim22 and Tim23 (all in 3.A.8.1.1).

^g^ Members of subfamily 3 of the PgaA family (1.B.55) show extensive sequence similarity with Tom70 (3.A.8.1.1) and Toc64 (3.A.9.1.1).

^h^ These three families were entered into TCDB after completion of the work described in this review.

Specifically, we could provide evidence that 47 of the 68 (putative) β-barrel OMPP families belong to a single superfamily, hereafter referred to as Superfamily I (SFI). Using the superfamily tree (SFT) program [12, 21, 35], we have drawn the first phylogenetic tree for the superfamily, revealing which of these families are likely to be most closely related. The results support the suggestion [31] that many families of OMPPs derive from a single common ββ-hairpin structure. We also confirm relationships suggested from family assignments in Pfam (see Table 1). However, indirect evidence is presented suggesting that some OMPPs do not derive from the same source. We also provide evidence for the existence of four small OMPP superfamilies, two in eukaryotic organelles, and two (α-TMS and β-TMS structural OMPPs, respectively) in Actinobacteria. The results reported extend suggestions made previously and put OMPPs in a phylogenetic framework.

## Methods

### Family and superfamily identification and characterization

In order to estimate relative family sizes, OMPPs of the 76 families in TCDB were used as query sequences for BLAST searches of the non-redundant NCBI protein database (default settings), which were conducted without iterations [38]. From one to 5,000 homologous proteins were retrieved from the NCBI database for each of the families, and these numbers were recorded in Table 1 to indicate the relative sizes of the families. Redundant and incomplete sequences were eliminated, and remaining selected proteins were retained for topological and phylogenetic analyses.

The CLUSTAL X program [39] was used with default parameters for multiple alignment of homologous sequences, and the TreeView [40] and FigTree programs [41] were used for the construction of phylogenetic trees for members of individual families. Alternative methods of tree construction, dependent on tens of thousands of BLAST bit scores and obviating the need for construction of a multiple alignment, were provided by the SuperfamilyTree (SFT) programs, SFT1 and SFT2 [21], [42], [35]. Previous publications have shown that these two programs give excellent agreement with trees derived using ClustalX/TreeView when sequences are sufficiently similar to generate reliable multiple alignments [21, 35, 42]. However, the SFT programs are superior when proteins with more divergent sequences are analyzed [11, 13, 43]. The SFT1 program shows the relationships of all proteins included in an analysis, while the SFT2 program shows the subfamily or family relationships within a superfamily.

Topological analyses of individual proteins were performed using the WHAT [44], HMMTOP [45] and Spoctopus [46] programs which we have shown are among the most reliable programs for topological predictions [47]. Average hydropathy, amphipathicity and similarity plots were generated using the AveHAS program [48]. PRED-TMBB [49] (http://bioinformatics.biol.uoa.gr/PRED-TMBB/) was used to predict numbers and positions of transmembrane β-strands for β-barrel proteins. HHrepID, [50] (http://toolkit.tuebingen.mpg.de/hhrepid#), a bioinformatics tool kit that uses HMM-HMM comparisons, was used to find structural repeats in protein sequences.

### Statistical approaches to homology establishment

Statistical sequence similarity comparisons between proteins, and between internal regions of these proteins, were conducted using the IC [36], GAP [45], Protocols 1 and 2 [51] and GSAT [36] programs. These programs randomly shuffle the sequences of the proteins or protein segments under scrutiny and compare these shuffled sequences with the native sequences. They thereby correct for abnormal protein compositions such as those that can occur in integral membrane proteins. Two thousand random shuffles and default settings have proven to be satisfactory for obtaining statistically significant values with both Protocol 2 and GSAT (see below). A comparison score of 12 standard deviation (SD) for comparable regions of two proteins of at least 60 amino acyl residues (aas) has been reported to correspond to a probability of 10^−27^ that the observed degree of sequence similarity arose by chance [52]. Although the actual probability may be much higher due to Gaussian skewing, this value has been considered sufficient to strongly suggest homology, given the NCBI protein database size when these studies were conducted [7].

### Obtaining homologues and removing redundancies

Query sequences used to identify members of OMPP families were taken from families 1.B.1 to 1.B.76 in TCDB. NCBI *PSI*-BLAST searches were conducted with two iterations (e^-4^; e^-6^ cutoff values, respectively). These searches were performed using Protocol1 [36] to identify members of each family. The Protocol1 program compiles homologous sequences from each BLAST search into a single file in FASTA format. It then eliminates redundancies and fragmentary sequences and generates a table of the resultant collection of sequences containing protein abbreviations, sequence descriptions, organismal sources, protein sizes, gi numbers, organismal groups or phyla, and organismal domains. Protocol1’s CD-HIT option was used to remove redundancies and highly similar sequences [36, 53]. An 85% identity cut-off was used to retrieve sequences that were subsequently used to establish homology between family members, and a 70% identity cut-off was used to create more easily viewed average hydropathy plots and phylogenetic trees. These percent identity values refer to the values above which all but one of the most similar sequences were removed. Thus, an 85% cutoff means that no two protein sequences retained for analysis were more than 85% identical. FASTA files from Protocol1 were considered representative of each respective protein family, although selected proteins that demonstrated apparent homology between families were sometimes confirmed with Pfam, NCBI’s Conserved Domain Database (CDD) [54], and PSI-BLAST [55] results as outlined above (see also Discussion).

### Multiple alignments and topological analyses

The ClustalX program was used to create multiple alignments of homologous proteins within individual families, and the few sequences that introduced large gaps into the alignment (usually a reflection of fragmentation, inclusion of introns or artifactual sequences) were removed. This allowed the generation of coherent multiple alignments where all or most sequences are homologous throughout most of their lengths. Results obtained with this program have been compared with 5 other programs, and when sequence similarity was sufficient to give reliable multiple alignments, phylogenetic trees obtained with the six programs (Neighbor Joining or Parsimony) were very similar [14]. The conserved domain database (CDD) (57) was also used to analyze protein sequence extensions that can result from the presence of extra protein domains as initially revealed using AveHAS plots [48].

### Establishing homology between families

Initially, a large screen was performed, comparing distantly related OMPP family members against members of all OMPP families (TC subclass 1.B) [36]. The Targeted Smith-Waterman Search (TSSearch) feature of Protocol2 was then run in order to compare each family to all other OMPP superfamily members. TSSearch uses a rapid search algorithm to find distant homologues within the two different FASTA files that may not readily be apparent from BLAST or PSI-BLAST searches [36]. The most promising comparisons between proteins were automatically analyzed using the Global Sequence Alignment Tool (GSAT) feature of Protocol2 [36]. Comparison scores obtained using GSAT are reported in standard deviations (SD). Scores were calculated with the Needleman-Wunsch algorithm [56]. Promising results with comparison scores of 12.0 SD or greater were confirmed and analyzed further using the GSAT program set at default settings with a gap creation penalty of 8 and a gap extension penalty of 2 with 2,000 random shuffles. Two families within TC subclass 1.B were initially excluded from our studies. These were the Autotransporter-2 (AT-2) Family (1.B.40) and the Intimin/Invasin (Int/Inv) Family (AT-3, 1.B.54). Many of these proteins have huge passenger domains of >1,000 aas with relatively small transmembrane β-barrel domains. Since the passenger domains frequently include β-structure, their presence complicated the assignment of homology, warranting their initial exclusion from our homology studies with other OMPP families. However, in subsequent studies, the transmembrane domains of these families were examined for tentative relationships with other families.

Comparison scores were calculated using Mathematica (Wolfram Research, Inc., Champaign, IL, USA). Comparisons involved protein segments of at least 60 amino acyl residues (aas), the average size of a prototypical protein domain, and required a comparison score of at least 12.0 SD to provide evidence for homology [57]. Convergent sequence evolution is possible and has been demonstrated for short motifs, but not for large segments of proteins such as entire domains. GSAT alignments were sometimes performed on sequences by taking segments of at least 60 aas, maximizing the number of identities, minimizing gaps, and removing non-aligned sequences at the ends of the alignment, but never in central regions of an alignment. Thus, all segments analyzed are derived from contiguous portions of proteins.

The Ancient Rep (AR) and GSAT programs [36] were used to identify internal repeats, and the HHRep [58] and HHRepID [50] programs provided independent search approaches. The AR program compares potential transmembrane repeat sequences (e.g., transmembrane regions predicted by HMMTOP) within a single protein and between proteins in a FASTA file, giving a comparison score in SD in the same format as Protocol2. The HHRep programs show graphical representations of similarities with repeat sequences revealed as lines parallel to the diagonal line representing the protein sequence itself. Results could often be confirmed using the MEME program [59] for conserved motif identification.

## Results

### OMPP families in TCDB

TCDB included 76 families of OMPPs in TC subclass 1.B at the time these studies were updated (5/2014), 62 of them being transmembrane β-barrel structures with varying numbers of transmembrane β-strands (β-TMSs), nine containing both α- and β-structure, and five consisting only of transmembrane α-structure (see Table 1). The large majority of these families (64) include members from Gram-negative bacteria, but six families are primarily from Actinobacteria, and 6 are primarily from eukaryotes. Table 1 summarizes characteristics of these 76 families as well as three additional OMPP families added more recently, while Table 2 summarizes the dominant phyla from which the members of these families derive, and Table 3 summarizes characteristics of the five superfamilies identified (see below). Column 1 in Table 1 presents the family TC numbers while column 2 presents the family names and their abbreviations. Column 3 lists the dominant organismal phyla from which these proteins are known to derive. Column 4 provides the average protein sizes ± SD, expressed in numbers of amino acyl residues (aas) for family members included in TCDB as of 5/2014. Column 5 gives the relative family sizes, estimated by the number of proteins retrieved in a single PSI BLAST search of the NCBI NR protein database without iterations when the first member of each family (1.B.X.1.1) was used as the query sequence. The maximal number of proteins retrievable in any one search was 5,000, so the few families reported to have this number of members are larger than indicated. Column 6 indicates the superfamily, if any, as defined in this paper, to which the family belongs. Column 7 presents the known or estimated numbers of transmembrane β-strands (β-TMSs) in protein members. An asterisk indicates that for one or more representative member(s), the 3-D structure is known, and consequently the topology of that protein is established. It should be noted that not all members of a family necessarily have the same number of β-TMSs. For those families lacking an asterisk, the numbers recorded were estimated using average hydropathy/amphipathicity/similarity (AveHAS) plots as well as the PRED-TMBB β-TMS prediction program for β-barrel proteins. In some cases, the proteins are known or predicted to consist of both α-helical and β-structural regions, and in these cases, we indicated this fact by “α + β”. Finally, the last column indicates the designation of the family or superfamily used by the Conserved Domain Database (CDD), often derived from the Pfam database. Although Table 1 is self explanatory, some of the features will be described below.

**Table 2:** Comparison of the predominant organismal types for the various OMPP families in superfamilies (SFI-SFV; column 3) with those not in superfamilies (column 4). Family TC #s are provided; Thus, 1.B.1 = 1; 1.B.2 = 2; 1.B.3 = 3; etc.

Superfamily	Dominant phylum	TC Family numbers in superfamilies	Families not in superfamilies
SF I	Proteobacteria	1, 3, 4, 5, 6, 7, 9, 10, 11,12, 13, 14, 15, 16, 17, 18, 19, 20, 21, 22, 25, 26, 31, 33, 39, 42, 43, 49, 54, 55, 57, 60, 61, 62, 66, 67, 68, 70, 71, 73	27, 40, 46, 48, 53, 63, 64, 65, 75, 76
	Proteobacterial totals	40 Families	10 Families
	Chlamydiae	2, 72	
	Mitochondria (Eukaryotes)	8	
	Cyanobacteria	23	
	Spirochaetes	35	36, 37, 38, 45, 51, 52, 56, 74
	Fusobacteria & Bacteroidetes	32, 44	
SF II	Actinobacteria	24, 58	41, 50
SFIII	Chloroplasts (Eukaryotes)	28, 47	29
SF IV	Eukaryotes	30, 69	
SF V	Actinobacteria	34, 59	
	Grand total	55	21

**Table 3:** Five superfamilies of OMPPs identified in this analysis. The table presents column 1, the superfamily number; column 2, the number of TC families in each superfamily; column 3, the relative superfamily size in numbers of proteins identified; column 4, the average protein size, expressed in numbers of amino acyl residues, ± standard deviations; column 5, numbers of superfamily proteins in TCDB as of 5/2014, and dominant organismal type represented.

Superfamily	# of TC Families	Superfamily Size (# proteins)	Average Protein Size (# aas)	Proteins in TCDB	Dominant Origin
I	47	76,760	471 ± 191	783	Proteobacteria
II	2	491	254 ± 62	11	Actinobacteria
III	2	139	277 ± 50	7	Chloroplasts
IV	2	499	199 ± 33	14	Eukaryotes
V	2	24	54 ± 15	16	Actinobacteria

As discussed in greater detail below, we have been able to assign many of the OMPP families to one large and four small superfamilies (Table 1, column 6 and Tables 2 & 3), which we have designated with roman numerals. Thirty three Pfam/CDD families, corresponding to 47 TC families, proved to fall into our Superfamily I, SFI; (Table 1). Each TC family usually corresponds to a distinct CDD family, although some TC families encompass more than one CDD family [60]. For example, TC family 1.B.6 (the OmpA/OmpF OMPP (OOP) Family) includes the OmpA, Omp β-brl and PRK10808 families of CDD; TC family 1.B.11 (the Fimbrial Usher Family) has four designations in CDD, PapCN, Usher, PRK15213 and PapCC. TC family 1.B.18 (the Outer Membrane auxillary (OMA) Family) has two designations in CDD, Poly Export and SLBB.

Some other TC families with multiple Pfam/CDD designations include families 1.B.4, 1.B.23, 1.B.38, 1.B.48 and 1.B.54. This last family has four distinct designations in CDD. Additionally, the CDD Omp β-brl superfamily is found as TC families 1.B.6 and 1.B.43. Seven TC families have no designation in CDD (Table 1), which merely reports “no putative conserved domains have been detected”. In such cases, column 8 in Table 1 is left blank.

OMPP families not recognized by CDD derive from a variety of organismal sources, and in general, they include low to moderate numbers of members. SFI families are derived almost exclusively from Gram-negative bacteria. CDD recognizes our Superfamily II (SFII) as the MspA Superfamily while a single family (1.B.47) of our superfamily III (SFIII) was recognized by CDD, but the other family of this superfamily (1.B.28) was not recognized. CDD recognized SFIV but not SFV. Establishment of the number of β-TMSs for one member of a family does not necessarily imply that all members of that family have the same topology as noted above. Finally, in several cases, some members of a TC family were recognized by CDD while others were not.

About two thirds of the TC OMPP families have their members derived primarily from Proteobacteria (Table 2). Nine families are derived primarily from spirochaetes, six from Actinobacteria, four from chloroplasts, two from chlamydiae, and one family each is derived from mitochondria (eukaryotes), peroxysomes (eukaryotes), fusobacteria, cyanobacteria and bacterioidetes (Table 2). Thus, eukaryotic OMPPs include four from chloroplasts, one from mitochondria and one from peroxisomes. It should be noted that this skewed distribution with so many families derived predominantly from Proteobacteria undoubtedly reflects in part the facts that so many proteobacterial genomes have been sequenced and so much experimental work has been conducted with these organisms. Although most firmicutes lack an outer membrane and therefore lack OMPPs, a few have been reported to have these structures, and these unusual firmicutes sometimes proved to encode OMPP homologues in their genomes [61].

### Establishing homology between families with the formation of superfamilies

**Fig 1:**
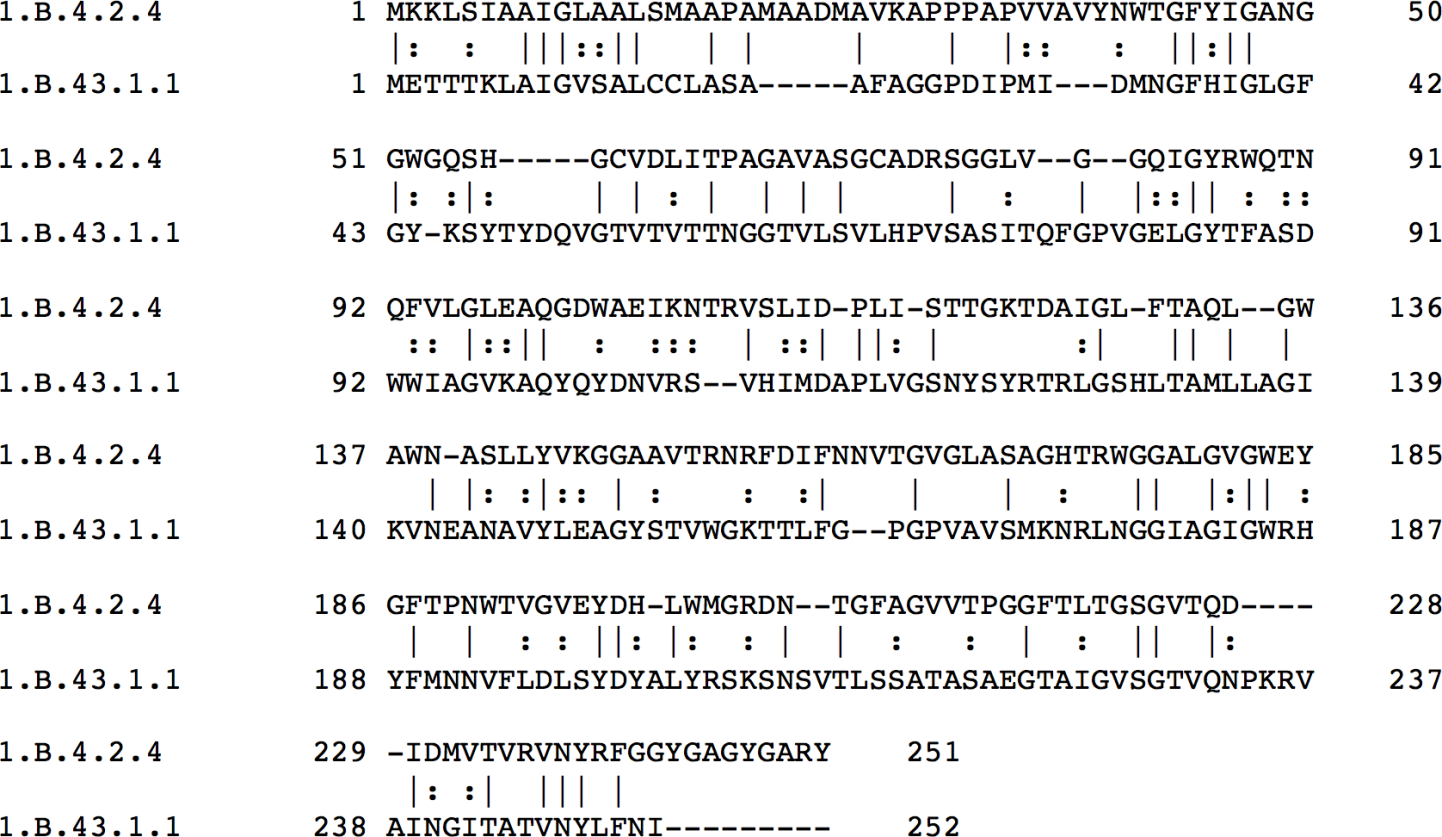
Binary alignment of a member of the BRP family (1.B.4) with a member of the CPP1 family (1.B.43). The alignment was generated with the GSAT program and shows the full sequences of both proteins. Residue numbers are indicated at the beginning and end of each line. A vertical line indicates an identity, and a colon indicates a similarity. The comparison score is provided in Table 4.

Members of the 76 OMPP families (TC subclass 1.B), were compared with each other using a variety of programs. First, the proteins of a family in TCDB were compared using TC-BLAST, which sometimes brought up members of other OMPP families. For example, when 1.B.4.2.4 was compared with 1.B.43.1.1, a binary sequence alignment was obtained that gave 26% identity and 44% similarity. Second, when binary comparisons looked promising, comparison scores were calculated using GSAT. For example, when GSAT was run with 2,000 random shuffles, for the two proteins noted above (4.2.4 and 43.1.1; the subclass, 1.B, will usually be omitted from the TC # from here on out when citing family, subfamily and protein TC #s), a comparison score of 15 SD was obtained, a value sufficient to establish homology. This alignment is shown in Fig 1 as an example. Third, if comparison scores were insufficient to strongly suggest homology, Protocol 1 was used to retrieve homologues of the two query sequences using NCBI PSI-BLAST with one or two iterations followed by comparison of all retrieved sequences in one list with those in the other list using Protocol 2 [36]. Fourth, top scores obtained with Protocol2 were confirmed using GSAT with 2,000 random shuffles. When adequate values were obtained, the two sequences compared by Protocol2 were then compared with the original query sequences from TCDB using GSAT with 2,000 random shuffles (Table 4). Only if all three values exceeded 12 SD did we conclude that evidence for homology was appreciable (see below). It should be noted that the inability to establish homology does not prove that a family is *not* a member of a superfamily.

**Table 4:** Comparison scores for TC families that comprise OMPP superfamilies I-III. Proteins 1 and 4 are the characterized family members in TCDB, while Proteins 2 and 3 were obtained using Protocol 1 and compared using Protocol 2 and GSAT (see Methods).

				Comparison Score (S.D.)			
Protein1 (A)	Protein2 (B)	Protein3 (C)	Protein4 (D)	A vs B	B vs C	C vs D	A vs D
1.B.1.3.4	Mha2	Vsp1	1.B.21.3.1	37	12	89	8
1.B.1.4.3	Rpa2	Hsp2	1.B.33.2.1	20	13	27	2
1.B.1.6.3			1.B.5.1.2				12^a^
1.B.1.1	Eba2	Pbe3	1.B.16.1.3	80	13	13	0
1.B.1.1.7	Sma2	Psp2	1.B.7.1.5	19	15	13	7
1.B.2.1.3	Sne2	Ppa1	1.B.9.4.1	179	12	17	3
1.B.3.1.6	Ppi1	Cje1	1.B.60.1.1	140	13	267	5
1.B.3.1.5	Pru2	Ppi1	1.B.16.2.3	132	166	12	8
1.B.4.2.1	Mau2	Pst1	1.B.6.2.8	30	20	15	10
1.B.4.2.4			1.B.43.1.1				15^a^
1.B.4.2.2			1.B.23.1.17				18^a^
1.B.4.2.1	Kgr1	Mpu1	1.B.9.2.2	42	13	66	10
1.B.4.2.3	Aca1	Pfl3	1.B.39.1.4	42	14	124	2
1.B.4.2.3	Abr6	Vch1	1.B.26.1.2	31	12	150	6
1.B.4.2.12	Csp1	Abr1	1.B.70.1.1	49	12	57	4
1.B.5.1.1	Gbe1	Mba1	1.B.16.2.3	12	37	21	1
1.B.5.1.2	Ofr2	Mgl1	1.B.62.1.2	16	13	18	4
1.B.5.1.9	Mgl1	Mam1	1.B.60.1.2	31	20	7	6
1.B.6.12	Wen1	Aph1	1.B.49.1.2	13	16	40	5
1.B.6.1.2	Wen1	Aph1	1.B.49.1.2	13	16	40	2
1.B.8	Chi1	Ssu4	1.B.23	51	13	13	7
1.B.10.2.2	Abe1	Plo1	1.B.33.5.1	43	13	21	3
1.B.11.3.4	Sfo10	Hbi1	1.B.42.1.6	96	13	12	7
1.B.12.8.3	Bau1	Lcr1	1.B.43.1.4	52	12	16	5
1.B.9.2.3	Nsa2	Cab7	1.B.57.4.6	24	15	52	9
1.B.13.1.3	Tye1	Mmi4	1.B.23.1.10	84	12	12	7
1.B.14.1.16	Pch1	Mgr1	1.B.9.2.1	135	25	22	4
1.B.15.1.5	Vga1	Vch2	1.B.1.1.10	39	13	19	7
1.B.16.1.3	Xca1	Lsp3	1.B.66.1.4	127	13	287	3
1.B.16.1.3	Msa1	Bba1	1.B.67.1.6	118	202	12	10
1.B.17.3.8	gi # 241554290	gi # 296396347	1.B.12.6.1	150	13	334	0
1.B.18.1.1	gi # 110633009	gi # 333812091	1.B.14.5.1	49	13	37	0
1.B.19.1.1	Aba2	Aur1	1.B.23.1,2	132	185	22	11
1.B.21.2.1			1.B.35.2.1				19^a^
1.B.22.3.1	gi # 323388142	gi # 154367880	1.B.9.2.3	222	13	170	1
1.B.23.1.4	Ssp10	Dsp4	1.B.62.1.1	108	18	29	11
1.B.23.1.4	Tna2	Dpr1	1.B.60.1.1	21	19	30	4
1.B.23.1.4	Tca1	Ccu2	1.B.31.1.4	43	12	23	7
1.B.24.1.2	Req1	Nbr4	1.B.58.1.2	21	35	54	11
1.B.28.1.3	Cca1	Ath1	1.B.47.1.3	13	12	98	11
1.B.30.3.1	Sst1	Dfa2	1.B.69.1.5	14	15	67	6
1.B.31.1.1	Hca1	Sde6	1.B.25.1.29	23	13	39	9
1.B.31.1.4	Abu4	Gsp1	1.B.61.1.2	113	13	50	6
1.B.31.1.4	Ccu2	Ote2	1.B.62.1.1	23	12	20	4
1.B.32.1.1	gi # 262066222	gi # 38569942	1.B.9.2.3	149	13	110	2
1.B.33.2.3	Tca1	Pva1	1.B.20.1.4	27	20	49	10
1.B.39.1.4	Sag1	Asu2	1.B.6.2.9	25	15	52	10
1.B.44.2.1	Pin1	Aki1	1.B.14.2.10	13	12	61	0
1.B.54.1.7	gi # 91069978	gi # 2622854505	1.b.22.3.2	174	14	159	0
1.B.55.1.2	Psp1	Cli2	1.B.14.12.3	53	37	49	5
1.B.60.1.1	Dal1	Gme1	1.B.62.1.1	30	12	16	0
1.B.66.1.2	Fag1	Cag1	1.B.71.1.6	31	12	12	5
1.B.68.1.1	Eco1	Tsu2	1.B.1.8.2	91	14	176	11
1.B.72.2.3			1.B.4.2.1				25^a^
1.B.73.1.3	Sfu2	Sru4	1.B.23.1.18	170	17	31	7

^a^ When scores for A vs. D were equal to or exceeded 12 S.D., as illustrated in Fig 1, the use of Protocol I and Protocol II to give comparison scores for A vs B, B vs C and C vs D, as illustrated in Fig 2A–2C, were not necessary.

**Fig 2:**
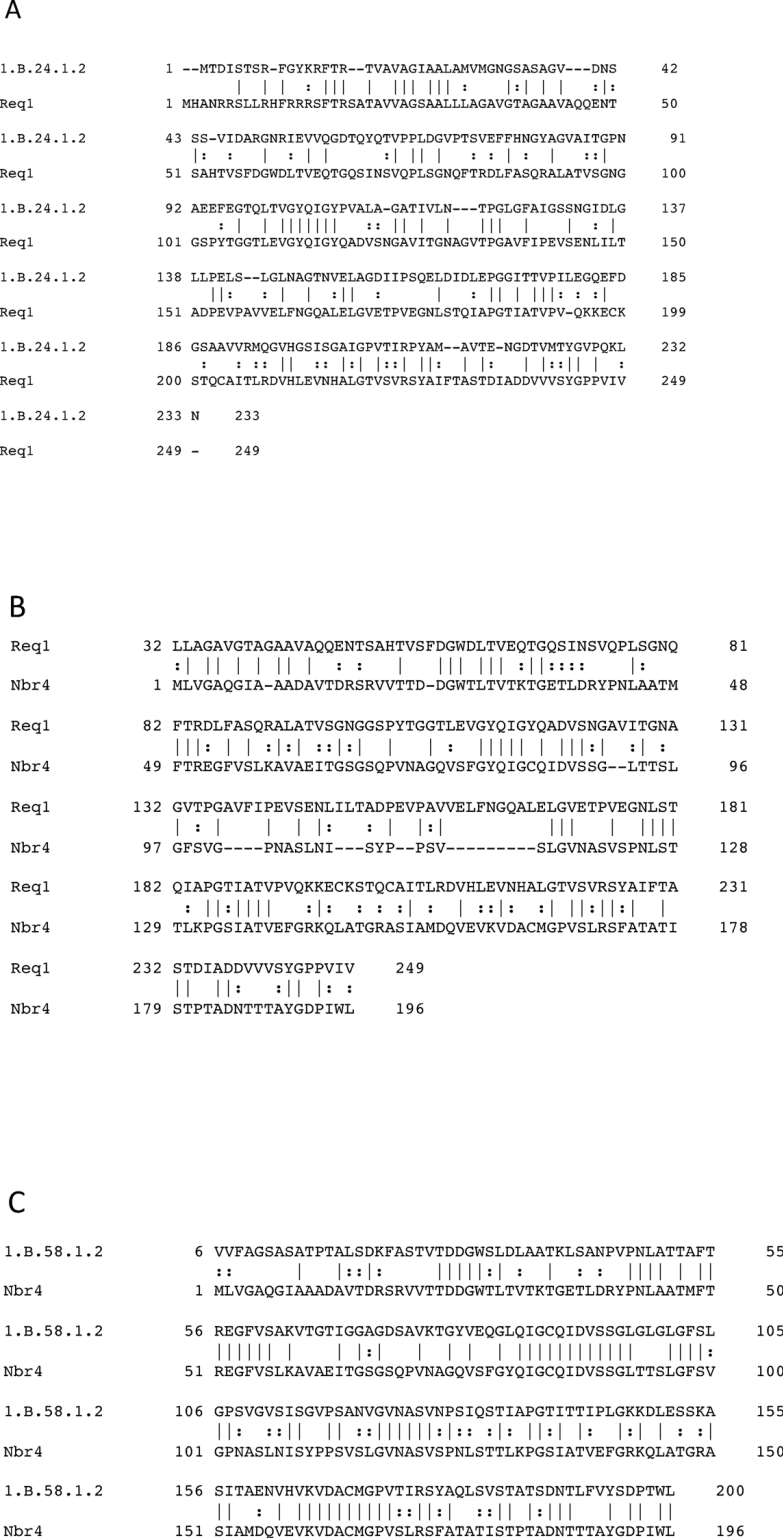
Illustration of the use of the Superfamily Principle to establish homology between two proteins in different families that have shown insufficient sequence similarity to allow demonstration of homology by direct comparison. A, B, and C show alignments of proteins A with B, B with C and C with D, respectively. Protein A, 1.B.24.1.2; Protein B, Req1, obtained with Protocol 1 with 1.B.24.1.2 as the query sequence. Protein C, Nbr4, obtained with Protocol 1 with 1.B.58.1.2 as the query sequence, Protein D; 1.B.58.1.2. Comparison scores are provided in Table 4.

An example of this procedure is shown in Fig 2A–2C, and the results of this comparison and others are summarized in Table 4. Protocol 1 retrieved Req1 when the query sequence was 1.B.24.1.2. The alignment obtained between these two proteins is shown in Fig 2A and gave a comparison score of 21 SD. The comparison of Req1 with a protein retrieved by Protocol 1 when 1.B.58.1.2 was the query sequence, Nbr4, is shown in Fig 2B. This comparison gave 35 SD. Finally, when Nbr4 was compared with 1.B.58.1.2, the alignment shown in Fig 2C was obtained, yielding 54 SD. When we compared 1.B.24.1.2 with 1.B.58.1.2 directly, the comparison score was only 11 SD, which is insufficient to establish homology by our criteria. The suggestion of homology using this approach depends on the Superfamily Principle. The results summarized in Table 4 provide the basis for the conclusion that our studies have defined the TC family compositions of Superfamilies I-IV. Comparable scores could not be obtained for SFV because of the small sizes (<60 aas) of these proteins (see Tables 1 and 3).

### OMPP Superfamily I (SFI)

**Fig 3:**
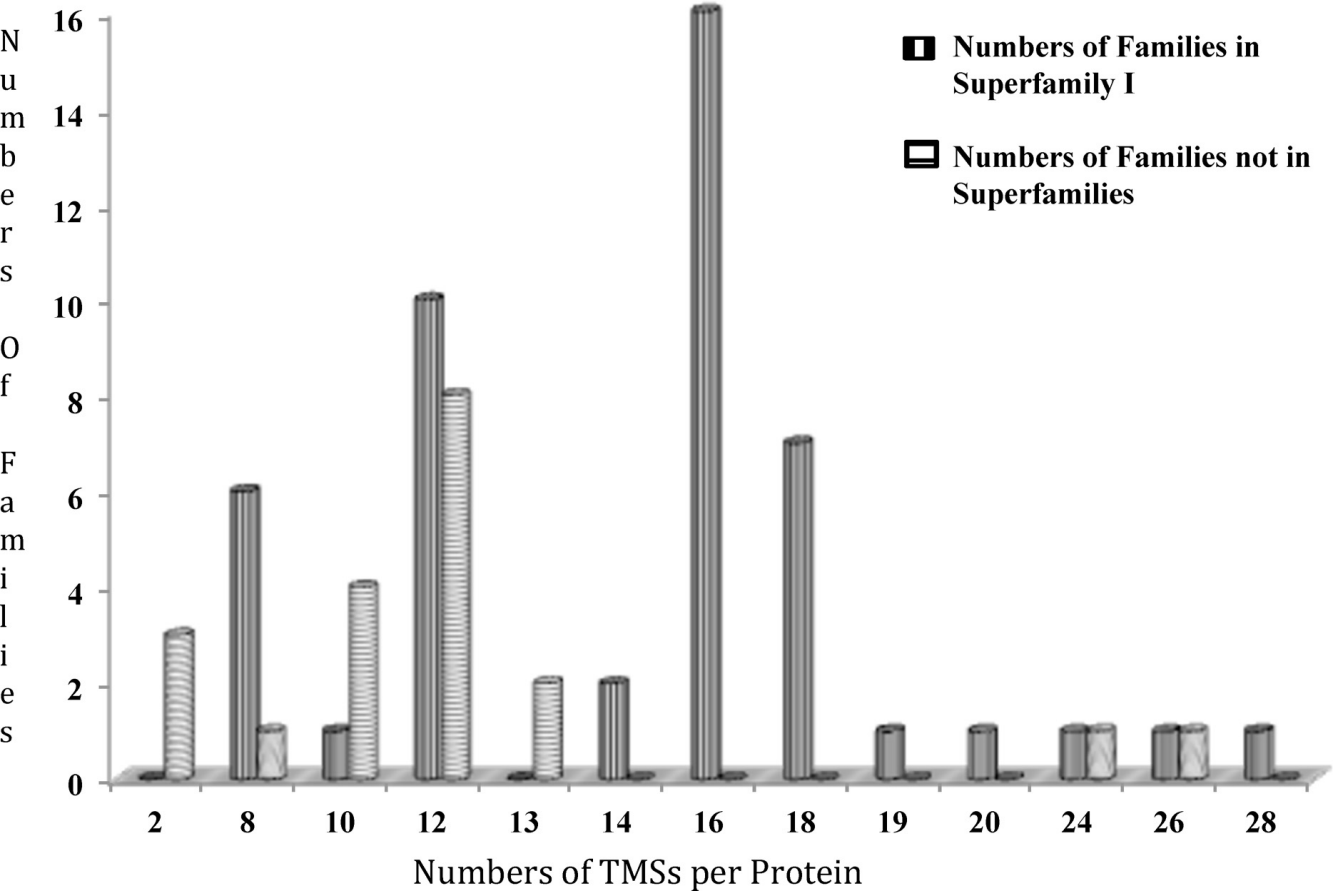
Topological comparison between proteins in Superfamily I ([] ) and those not in a superfamily (| = |). A single established or predicted topology is included for each family, although it is possible that some families include members with more than one topology (see Table 1).

We have identified five superfamilies, each of which includes at least two TC OMPP families (Tables 3 and 4). TC Superfamily I consists of forty seven TC families and is by far the largest and most diverse. The number of proteins within this superfamily in the NCBI protein database as of 10/2014, was over 50,000, obtained by PSI-BLAST searches of the NCBI protein database without iterations. These proteins have an average size of 471 ± 191 amino acids, showing a very substantial degree of size variation. The proteins represented in this superfamily can have any of the following putative or established numbers of β-TMSs within the putative barrel: 8 TMSs (1.B.6; 1.B.39; 1.B.43; 1.B.44; 1.B.49; 1.B.68), 10 TMSs (1.B.66); 12 TMSs (1.B.4; 1.B.10; 1.B.12; 1.B.17; 1.B.22; 1.B.35; 1.B.54; 1.B.57; 1.B.71; 1.B.72), 14 TMSs (1.B.9; 1.B.32), 16 TMSs (1.B.1; 1.B.2; 1.B.5; 1.B.7; 1.B.15; 1.B.16; 1.B.18; 1.B.19; 1.B.20; 1.B.21; 1.B.23; 1.B.26; 1.B.31; 1.B.33; 1.B.67; 1.B.70), 18 TMSs (1.B.3; 1.B.13; 1.B.25; 1.B.60; 1.B.61; 1.B.62; 1.B.73), 19 TMSs (1.B.8), 22 TMSs (1.B.14), 24 TMSs (1.B.11); 26 TMSs (1.B.42) and 28 TMSs (1.B.55) (see Fig 3 for a graphical representation). Thus, we see that the order of frequency of topological types in SF I (# of β-TMSs) is 16 (16 families) > 12 (10 families) > 18 (7 families) > 8 (6 families) > 14 (2 families) > 10 = 19 = 22 = 24 = 26 = 28 (1 family each) (see Fig 3). Since 16 β-TMS proteins could have arisen by duplication of 8 β-TMS proteins, it appeared possible that the most common topological type (16 β-TMSs) observed in this superfamily, arose by duplication of 8 β-TMS precursor. This has been suggested previously on the basis of the properties of artificially constructed 16 β-strand OMPPs, generated by intragenic duplication of 8 β-strand OMPPs [62]. It should be noted that our attempts to document such a duplication using our statistical methods were unsuccessful.

Fig 3 also shows the numbers of established or predicted β-TMSs in the β-barrel OMPPs not in a superfamily. Interestingly, two of the three most common topologies observed for SFI (16 and 18 β-TMSs) are not represented at all among the non-SF families, and while 6 families of SFI have 8 β-TMSs, only one of the non-SF families have this topology. Other striking differences can be seen (Fig 3). These differences in topologies between proteins within SFI and those excluded from a superfamily suggest fundamental differences between these two sets of OMPPs (see Discussion).

### OMPP Superfamilies II-V (SFII-V)

Superfamily II (SFII) consists of two TC families with an average protein size of 254 ± 62 aas. The two families present in Superfamily II, 1.B.24 and 1.B.58, are both derived from Actinobacteria. Family 1.B.24 OMPPs are derived from Mycobacteria, while 1.B.58 OMPPs are derived from these and other actinobacterial genera including *Nocardia*. This superfamily is predicted to consist predominantly of 8 β-TMS (1.B.58) and 16 β-TMS (1.B.24) proteins. Thus, a primordial nocardial-type OMPP could have served as the precursor for mycobacterial-type OMPPs such as MspA via intragenic duplication, although we could not verify this suggestion using our statistical methods.

Superfamily III (SFIII) includes two TC families with an average protein size of 277 ± 50 amino acids. The two families included in SFIII are 1.B.28 and 1.B.47, both derived from plant chloroplasts. Family 1.B.28 OMPPs are predicted to have 12 TMSs while family 1.B.47 OMPPs are predicted to have 14 TMSs.

Four small OMPP families derived from Actinobacteria, and two families from eukaryotes have been extensively characterized (see reference citations in TCDB under each family), and they apparently consist primarily of α-TMSs. The latter two OMPP families (1.B.30 and 1.B.69) belong to superfamily IV and appear to have 4 α-TMSs per OMPP. They are in the Tim17 family of Pfam and are included in a more extensive superfamily in TCDB (see the TCDB Superfamily hyperlink for a list of other proteins included in the Tim17 Superfamily). Two of the actinobacterial families comprising superfamily V have been shown to be related, and they include small proteins, usually of 40–60 aas, with a single α-TMS (see Table 1). These two families are the PorA Family (TC# 1.B.34) and the PorH Family (TC# 1.B.59) (Table 1). These can either be hetero- or homo-oligomeric [63]. Because of their small sizes and the substantial sequence divergence of these two families, these proteins did not allow construction of reliable phylogenetic trees. In an independent study, we have shown that these two families consist of homologous proteins. In addition to being called OMPP SFV, they have been designated the Corynebacterial PorA/PorH Superfamily (T. Su and M.H. Saier; unpublished results; see TCDB Superfamily hyperlink). This superfamily will not be discussed further here.

An additional actinobacterial OMPP family including proteins of α-structure is the Corynebacterial PorB Family (TC# 1.B.41) [64]. A high resolution (1.8 Å) x-ray structure of the *Corynbacterium glutamicum* PorB monomer is available, revealing a globular bundle of 4 α-helices tied together by a disulfide bond [65]. The native membranous structure must be oligomeric to form a pore, and a model for such a structure has been proposed [65]. PorC homologues of Corynebacteria [64] are members of this family, but PorB/PorC homologues are not believed to be related to PorA/PorH proteins.

### OMPPs not included in superfamilies

Twenty-one families of OMPPs in TCDB did not fall into one of the five superfamilies mentioned above. Twenty of these families consist of proteins forming β-barrels that exhibit predicted topologies with any of the following numbers of putative β-TMSs within the barrel, based on PRED-TMBB: 12 (8 families) >10 (4 families) > 2 (3 families) > 13 (2 families) > 8 = 24 = 26 (1 family each). It is interesting to note that the distribution of topological types observed for these families is strikingly different from that observed for SFI (Fig 3). For example, three non-SF OMPP families have members with 2 predicted β-TMSs and presumably form oligomeric structures, but such proteins are not found in SFI. Moreover, the predominant predicted topologies are 12 > 10 β-TMSs, and none of these families appears to consist of proteins with 16 or 18 β-TMSs, two of the most common topologies observed for SFI. These observations suggest that there is a basic difference between families included in Superfamily I, and those not included in a superfamily. Possibly, these differences in topological distribution reflect a fundamental difference in their evolutionary pathways, which could suggest that all β-TMS OMPPs do not belong to a single superfamily (see Discussion).

### Topological analysis of the 76 TC OMPP families

**Fig 4:**
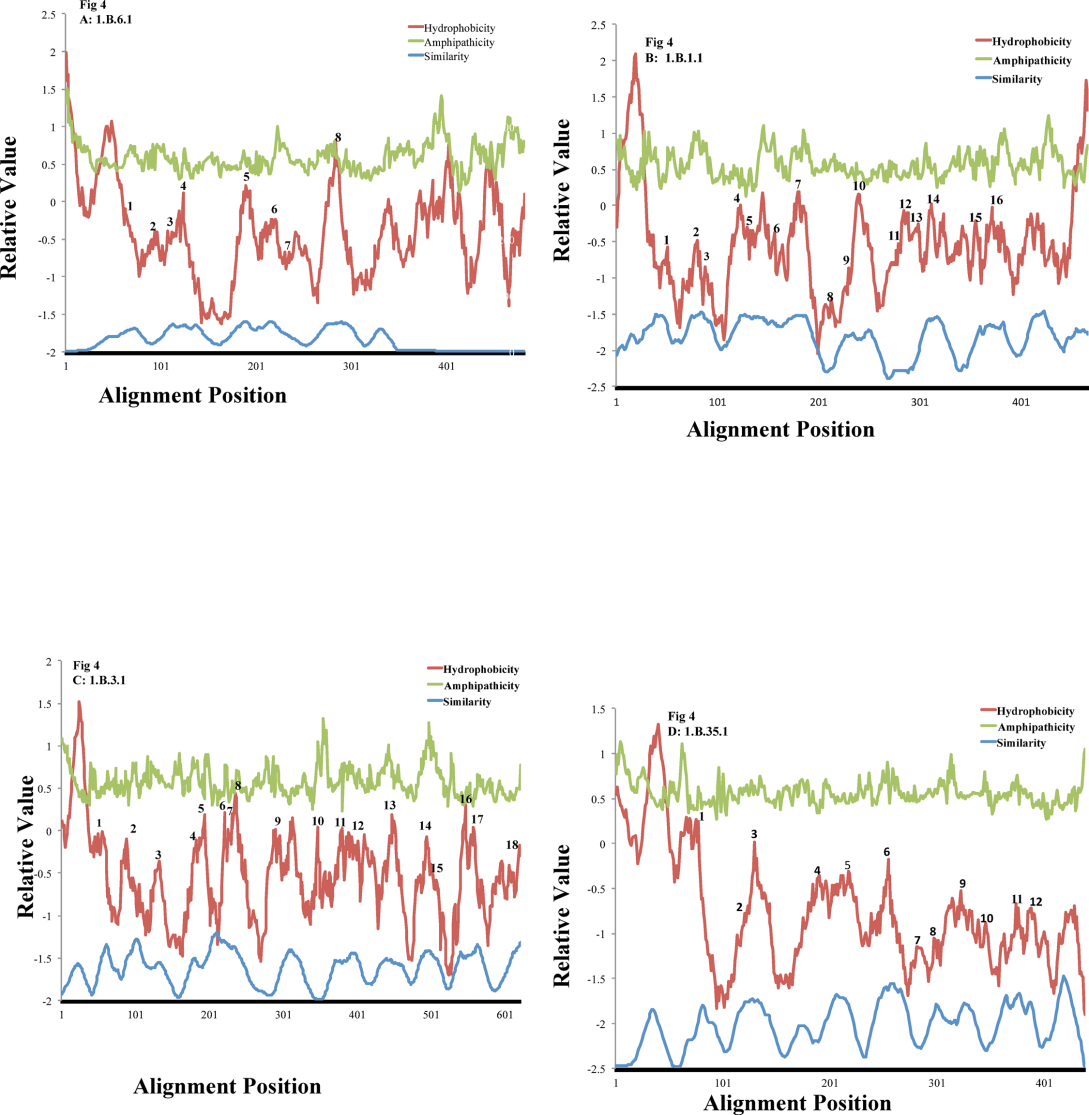
AveHAS plots of representative OMPP families showing upper light line, average amphipathicity; upper dark line, average hydropathy, and lower light line, average sequence similarity. Numbers above the hydropathy plot indicate the known positions of the β-strands. (A) the OOP Family (TC #1.B.6.1); (B) the GBP Family (1.B.1.1); (C) the SP Family (1.B.3.1); and (D) the KdgM Family, (TC #1.B.35.1). β-TMS positions are based on high resolution X-ray crystallographic structures of representative family members. The alignments upon which these plots were based included all proteins within the indicated subfamily in TCDB. Note the correlation between the peaks of hydropathy (middle plots), and the peaks of sequence similarity (lower plots).

All 76 OMPP families were examined in three ways using the AveHAS and PRED-TMBB programs. First, the proteins of each family in TCDB were used to construct multiple alignments using the ClustalX program followed by input of the alignment into the AveHAS program for topological prediction. Second, homologues of the family were retrieved using the first protein in each TC family (1.B.X.1.1) as the query sequence in a single Protocol 1 search of the NCBI NR protein database with a cut off value of 80% using PSI-BLAST without iterations. Proteins obtained were again multiply aligned, and topology was again predicted using the AveHAS program. Third, several randomly chosen individual proteins in each family were examined using the PRED-TMBB program. A consensus value was then obtained and recorded in column 7 of Table 1. When 3-D structural data were available (values in column 7 of Table 1 marked with asterisks), the known topology was compared with the predictions, leading to the observation that these predictions were about fifty percent accurate. However, when several homologues of a single family are examined and the results are averaged, much greater accuracy can be attained. Examples are shown in Fig 4A–4D for subfamily 1.B.6.1 with 8 β-TMSs (Fig 4A), subfamily 1.B.1.1 with 16 β-TMSs (Fig 4B), subfamily 1.B.3.1 with 18 β-TMSs (Fig 4C) and subfamily 1.B.35.1 with 12 β-TMSs (Fig 4D). Because the 3-D structures are known for representative members of these four families, we could assign β-TMSs with high confidence. If the topology is the same for all included members of each subfamily, an assumption that may or may not be valid, depending on the family, this conclusion applies to all members of the subfamily. Thus, the numbers above the peaks of hydrophobicity in Fig 4A–4D give the positions of the known TMSs.

### Fusion of OMPPs with other OMPPs and other protein domains

OMPPs only rarely appear to be fused to other OMPPs or to other protein domains, but several examples were identified. For example, we identified a protein (1.B.4.2.2) that proved to be two OMPPs fused together. This protein is designated the high affinity Mn^2+^ OMPP, MnoP of 675 aas [66]. It has an N-terminal domain homologous to members of the *Brucella-Rhizobium* OMPP Family (BRP; 1.B.4), corresponding to the CDD Omp_b-br1 family, and a C-terminal domain homologous to members of the Cyanobacterial OMPP Family (CBP; 1.B.23), corresponding to the CDD OprB OMPP Family. These and similar fused OMPP proteins are found exclusively in α-proteobacteria, but they are present in many genera, including species of Rhizobia, *Rhodopseudomonas*, *Nitrobacter*, *Afipia* and *Oligotropha*. Interestingly, members of the CBP family have a fused N-terminal S-layer (SLH) domain [67] of about 100 residues, characteristic of this OMPP family.

A second protein (1.B.4.1.1) consists of 1115 aas and contains three OMPP domains. Residues 1–300 are homologous to OMPPs in family 1.B.4 discussed above, residues 350 to 840 are homologous to OMPPs of the Alginate Exporter OMPP (AEP) Family (1.B.13) which overlaps with the DUF4104 domain in CDD, and residues 860 to 1115 are again homologous to proteins in family 1.B.4. Thus, these three complete OMPP domains comprise the entirety of this large protein. A very similar fusion protein, lacking the first of these domains, but possessing the second two, was identified and assigned TC number 1.B.4.1.2. The two domains in this protein are 60% identical to the last two domains in 1.B.4.1.1. These proteins are found only in closely related α-proteobacteria such as species of *Methylosinus* and *Methylocystis*. We emphasize that all of these fusion proteins were found in α-proteobacteria, some of which are known to form protein fusions and domain rearrangements with higher frequency than other proteobacteria [15].

Additional fusion proteins were found in subfamily 2 of family 1.B.72, the Protochlamydial OMPP (PomS/T) Family. Members of subfamily 1 in this family include characterized chlamydial OMPPs [68]. The two proteins exhibiting fusions are (1) 1.B.72.2.3 of 577 aas which consists of a full length N-terminal BRP Family domain and a C-terminal PomS/T Family domain, and (2) 1.B.72.2.4 of 1086 aas which consists of a long N-terminal sequence including tetratricopeptide repeats and a C-terminal PomS/T domain. Interestingly, both of these sequences are also from α-Proteobacteria, and surprisingly, the PomS/T domain shows limited sequence similarity to members of the Omptin (Protease 7) Family. It is possible that Omptin Proteases (9.B.50), for which high resolution x-ray structures are available [69], are related to PomS/T OMPPs.

### Superfamily I phylogenetic analyses

**Fig 5:**
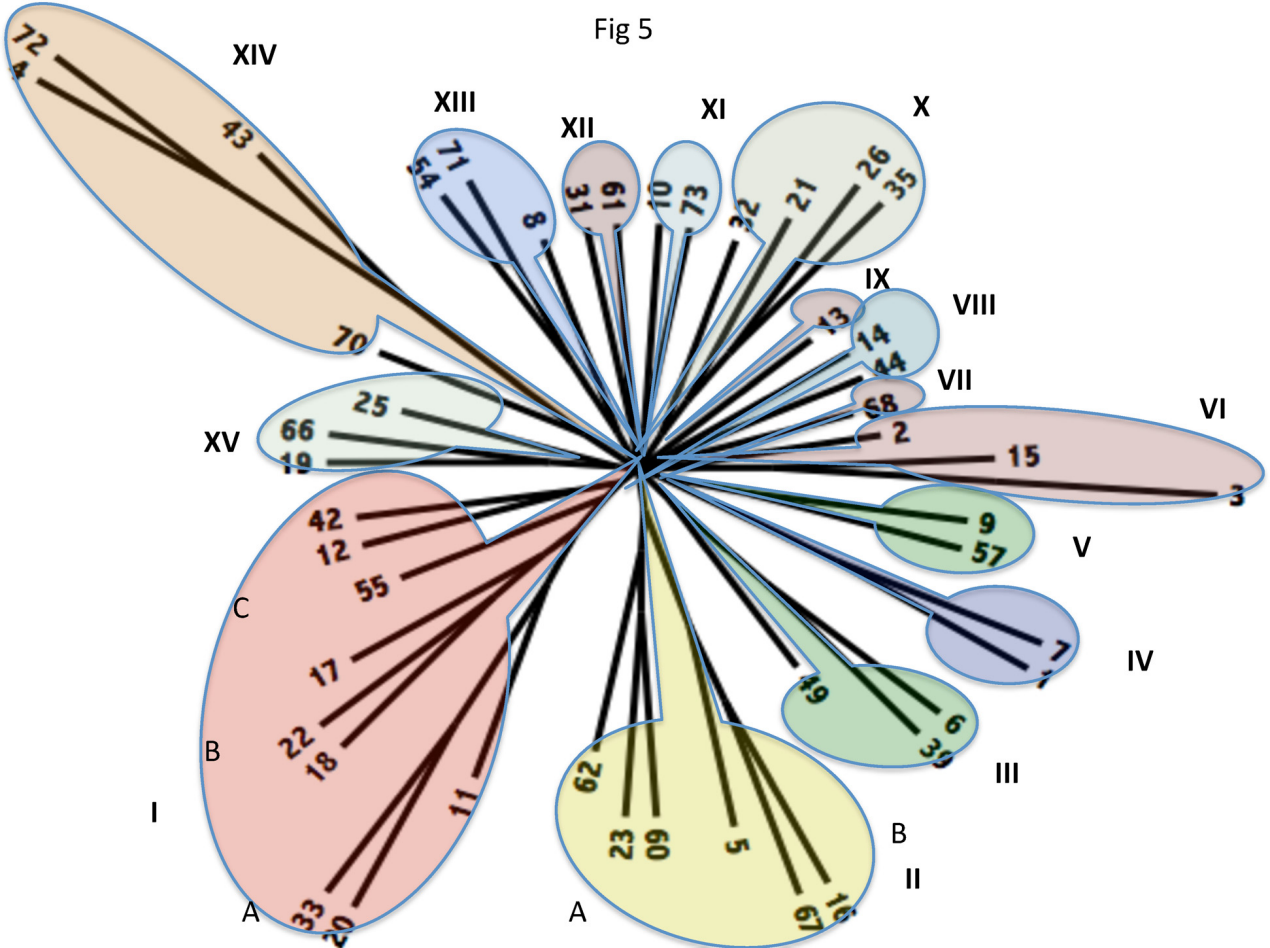
Phylogenetic tree based on the SuperfamilyTree programs (SFT1 and SFT2) for Superfamily I, showing the estimated family relationship based on tens of thousands of BLAST bit scores and the consensus of 100 trees.

Phylogenetic trees were constructed in six different ways. First, all or many representative proteins from a family within TCDB, included within any one of the 47 families that comprise Superfamily I, were used to generate a multiple alignment using the ClustalX program followed by tree construction using TreeView or FigTree, two equivalent methods. Second, the same method was used with a smaller collection of representative proteins from each of the TC families included in the study. Third, the SuperfamilyTree 1 (SFT1) program was used with all members of the families in TCDB included in the Superfamily I input file. Fourth, the same program was used with a single representative member of each subfamily in Superfamily I. In this last tree, we selected the first members of all subfamilies (1.B.X.X.1). Fifth, the SFT1 program was used with several representative proteins from each Superfamily I family. These studies revealed that for accurate tree construction, several members of a family (at least 5), must be included to obtain accurate relationships. Finally, the SFT2 program was used to derive a consensus tree using all of the data from SFTI (Fig 5). This tree shows the positions of the families relative to each other, revealing their probable relationships. See Tables 1 and 5 for details of these families, their properties and their relationships to each other. Table 5 presents the proposed phylogenetic relationships, functions, substrates when known, protein sizes, and (putative) topologies.

**Table 5:** SFI families included in clusters I-XV arranged according to the cluster/subcluster as shown in Fig 5. A bracket ({) indicates that these families are most closely related within the indicated (sub)cluster. See footnotes for explanation of the columns.

Cluster	TC #^1^	Family^2^	Substrates^3^	Average^4^ Size	Topology^5^
IA	{20	TPS	Proteins	589 ± 60	16
	{33	OmpIP	Proteins	594 ± 229	16
	11	FUP	Proteins	830 ± 32	24
1B	{18	OMA	Polysaccharides	393 ± 82	1 α Helix
	{22	Secretin	Proteins18	555 ± 151	12
	17	OMF	Proteins	472 ± 30	12
	55	PgaA	Polysaccharides	786 ± 155	28
1C	12	AT–1	Proteins	1345 ± 15	12
	42	LPS-EP	Lipopolysachaides	750 ± 223	26
IIA	{23	CBP	General	457 ± 56	16
	{60	Omp50	General	540 ± 49	18
	62	PBP	?	505 ± 71	18
IIB	{16	SAP	Amides, Urea	414 ± 32	16
	{67	BBP4	?	400 ± 45	16
	5	POP	Anion-selective	429 ± 58	16
III	{6	OOP	General	264 ± 85	8
	{39	OmpW	General	227 ± 10	8
	49	A-P44	Amino acids, sugars, oligosaccharides	266 ± 30	8
IV	1	GBP	General	347 ± 51	16
	7	RPP	General	333 ± 32	16
V	9	FadL	Hydrophobics	434 ± 34	14
	57	LM-OMP	?	370 ± 52	12
VI	{3	SP	Oligosaccharides	453 ± 48	18
	{15	RafY	Oligosaccharides	377 ± 63	16
	2	CP	Sugar, acids, etc.	328 ± 60	18
VII	68	BBP5	?	188 ± 7	8
VIII A; VIII B	14	OMR	Fe-Siderophores, Vitamins, etc.	235 ± 26	8
	44	PorT	Proteins	782 ± 135	22
IX	13	AEP	Polysaccharides	507 ± 96	18
X	{26	CDP	Polysaccharides	353 ± 27	16
	{35	KdgM	Polysaccharides	249 ± 21	12
	21	OmpG	General	316 ± 39	14
	32	FomP	General	331 ± 32	14
XI A; XI B	10	Tsx	Nucleosides	278 ± 13	12
	73	CBA	Polysaccharides	514 ± 50	18
XII A; XII B	31	MomP	General	456 ± 13	18
	61	Delta-OMPP	?	418 ± 41	16
XIII	{54	AT-3	Proteins	793 ± 572	12
	{71	PVP	?	247 ± 18	12
	8	MPP	General	290 ± 14	19
XIV	{4	BRP	General	266 ± 99	12
	{72	PomS/T	General	444 ± 243	12
	43	CPP1	General	242 ± 45	8
	70	OMC	?	468 ± 59	16
XV	{19	OprB	General	459 ± 63	16
	{66	BBP2	?	415 ± 50	18
	25	Opr	General	428 ± 44	18

^1^Family TC # in subclass 1.B. (see Table 1).

^2^Family abbreviation; see Table 1 for full name.

^3^Substrates shown to be transported by members of the indicated OMPP families.?, substrates unknown.

^4^Average protein size is provided in numbers of amino acyl residues ± standard deviation (SD).

^5^Topology expressed in numerical values refers to the established or predicted numbers of β-strands in the transmembrane β-barrel. All Cluster I OMPPs contain both α-helical and β-strand structures.

ClustalX–derived trees (e.g. S1 Fig) revealed clustering patterns that were inconsistent with the known phylogenies of the proteins. For example, the coherent but sequence diverse family of outer membrane receptors (OMR; 1.B.14) showed a majority of the members in one large cluster, but sequence divergent members of this family were found in eight additional clusters around the tree. Another large family, the Outer Membrane OMPP Family (Opr; 1.B.25), showed a majority of the members in a single large cluster, but other members appeared in three more clusters. The large General Bacterial OMPP Family (GBP; 1.B.1) had most of its members clustering in two large groups, separate from each other. Few families had all members correctly clustering together, as was true of the OprB Family (1.B.19), but these were the exception. In general, members of the large diverse families did not show consistent clustering, although members of the small sequence similar families sometimes did.

In other ClustalX based trees, where select sequence divergent proteins were included, a similar situation existed. When all members of a family selected for inclusion were derived from a single subfamily, the proteins frequently clustered together. This was true for families 7, 19, 31, 39 and 47. Only in one case (family 9), where the members selected were from different subfamilies, did they still cluster together. These results reveal the limitations of trees based on multiple alignments when sequence divergence is considerable as has been noted before [70].

### The SFT phylogenetic tree for Superfamily I

Using the SFT1 program, we first included all TC proteins within all of the 47 Superfamily I families. Clustering patterns were in general consistent with family assignments (see below), but the tree was so congested that it was impossible to display all of the proteins included. We next created a tree using only the first member of each subfamily within all families of the superfamily. This tree did not show the expected clustering of subfamilies within specific families, showing that it was necessary to include a substantial number of closely related proteins in order to generate a reliable tree. Consequently, we generated a final tree in which five proteins from each family within the superfamily were included. This tree proved to have members of each family generally clustering together with few exceptions. Thus, very few proteins fell outside of the cluster representing the family to which these proteins belonged. The tree, showing family relationships (Fig 5), derived using the SFT2 program, will be described in detail below.

The phylogenetic tree shown in Fig 5 includes fifteen major clusters, labeled I–XV. Most of these clusters include multiple families, although clusters VII and IX include only one family each. The clustering pattern reveals which families within Superfamily I are most closely related. Clusters IV, V, VIII, XI and XII contain two families each, clusters III, VI, XIII and XV have three families each; clusters X and XIV each have four families, cluster II has six families, and cluster I includes nine families. The families included in the 15 clusters are shown in Fig 5, and their properties are summarized according to cluster and subcluster (A, B and C) in Table 5.

Cluster I has three primary subclusters; subcluster A includes families 20 (The Two Partner Secretion (TPS) Family), 33 (The Outer Membrane Protein Insertion OMPP (OmpIP) Family), and 11 (The Outer Membrane Fimbrial Usher OMPP (FUP) Family). TPS and OmpIP OMPPs are most closely related, with the FUP Family branching from a point closer to the center of the tree. All three families include members that are derived from a variety of Gram-negative bacteria, especially proteobacteria (Table 1). Although as noted below, members of the OmpIP Family are present in mitochondria and chloroplasts, the organismal types cited in Table 1 represent those included in TCDB. At least in some of these families, other phyla are represented in the UniProt and GenBank databases.

Both of the first two families in subcluster A include C-terminal 16 stranded β-barrel OMPPs as well as additional domains that function in the insertion of outer membrane proteins (OmpIP), or the export of proteins across the outer membrane (TPS) [71, 72]. In both cases, the substrate protein folds into its native configuration during or soon after the export process [73]. It is interesting that the functional Omp85 (YaeT, BamA) OMPPs of the OmpIP family are related to the chloroplast import-associated β-barrel channel proteins (IAP75; 1.B.33.2.1) of the Chloroplast Envelope Protein Translocase (CEPT or Tic Toc) Family (TC# 3.A.9), and the Mitochondrial Sorting and Assembly Machinery (SAM) OMPPs, SAM50 (TC# 1.B.33.3.1), which assembles outer mitochondrial membrane β-barrel proteins [74–76]. As for the TPS OMPPs, the N-terminal domains of Omp85 homologues are localized to the periplasm, where they function in substrate protein binding and pore gating, while the C-terminal domains comprise the 16-stranded β-barrels [77, 78]. Interestingly, signals in bacterial Omp85 homologues are functional in eukaryotic cells for targeting to and assembly of mitochondrial OMPs into the outer membranes of these organelles [79].

The third family in subcluster IA with the TPS and OmpIP OMPPs, is the Fimbrial Usher Protein (FUP) Family (1.B.11). These large usher proteins resemble the TPS and OmpIP OMPPs in having extra N-terminal domains involved in substrate protein recognition as well as C-terminal extracellular domains that function in fimbrial subunit folding and assembly. In this case, the OMPP domain is central and has about 24 β-TMSs [80, 81]. Fimbrial ushers serve essentially the same function as TPS systems in exporting proteins, in this case, for assembling the subunits of bacterial fimbriae. They evolved in parallel with the periplasmic chaperone proteins that feed the subunits to the usher proteins [16].

The second major subcluster in cluster I, subcluster B, includes the Outer Membrane Auxillary (OMA) family of capsular polysaccharide exporters (1.B.18) and the bacterial Secretin (Secretin) Family (1.B.22), usually involved in protein secretion (most closely related), as well as the Outer Membrane Factor (OMF) Family (1.B.17) (more distantly related), involved in the export of extracellular proteins and polysaccharides as well as small molecules such as drugs, aromatic acids and divalent metal ions, depending on the inner membrane transport systems with which the proteins of this family associate [82, 83]. Like subcluster A, subcluster B is concerned with macromolecular export. Both the OMFs and the Secretins have proteins with α- and β-structure with 12–16 β-TMSs, and both form oligomeric structures [84]. The octomeric transmembrane ring of the OMAs has been compared with that of Secretins [85], although their transmembrane domains are formed of unusual α-helical barrels with three layered ring domains of mixed composition, mainly of β-strands in the periplasm [86].

Near the base of subcluster B, is the Poly Acetyl Glucosamine OMPP (PgaA) Family (1.B.55), another family concerned with extracellular polysaccharide export [87]. These OMPPs have the structure of a β-barrel with variable numbers of predicted β-strands.

At the base of cluster I is a subcluster (subcluster C) consisting of two families, the Autotransporter-I (AT-1) Family (1.B.12) and the Lipopolysaccharide Export OMPP (LPS-EP) Family (1.B.42). Like all other OMPPs in cluster I, these OMPPs are concerned with macromolecular (protein and carbohydrate, respectively) export. These two families cluster more closely to each other than to any other family in cluster I. While representative members of the AT-1 family are known to have 12 β-TMSs in β-barrel structures, the topology of the LPS-EP family is not known. It is remarkable that the nine OMPP families that comprise cluster I all serve a unified function in macromolecular export, particularly because few OMPP families include members with this capability. They also display the properties of being multi-domain multi-subunit systems in most cases.

Cluster II includes families that fall into two subclusters, each containing three families. Subcluster A includes the Cyanobacterial Trimeric OMPP (CBP) Family (1.B.23) [67], found in many bacterial phyla, the OMP50 OMPP (Omp50) Family (1.B.60) [88], represented in several Gram-negative bacterial phyla (these two families are more closely related) and the Putative Bacterial OMPP (PBP) Family with no functionally characterized members (1.B.62). The second subcluster in cluster II, subcluster IIB, includes the Short Chain Amide and Urea OMPP (SAP) Family (1.B.16), the Putative β-Barrel OMPP-4 (BBP4) Family (1.B.67) of unknown function, and the *Pseudomonas* OprP OMPP (POP) Family (1.B.5) of anion-selective OMPPs [89, 90]. The former two OMPP families are more closely related to each other than to the POP family. All cluster II families consist of members in about the same size range (400–540 aas) and are present in many different Gram-negative bacterial phyla. Moreover, all are predicted to have 16–18 β-TMSs arranged in β-barrels. The close relationships of the functionally uncharacterized PBP and BBP4 families with the four functionally characterized families provide the strongest evidence currently available that these two families do, in fact, consist of OMPPs. In contrast to cluster I OMPPs that have the capacity to export macromolecules, all characterized cluster II OMPPs apparently function to allow transport of small molecules.

Cluster III consists of three large families, all of which have small porin domains of about 200–250 aas with 8 established (2 families) or putative (1 family) β-strands in a barrel structure. However, several of these proteins are larger due to fusions with domains such as the peptidoglycan binding domain in several OmpA proteins [91].

The best characterized of these families are the OmpA-OmpF OMPP (OOP) Family (1.B.6) [92, 93] and the OmpW Family (1.B.39) [94]. The third family in cluster III is the *Anaplasma* P44 (A-P44) Family (1.B.49) with established OMPP activity [95]. Members of a family of spirochaete proteins, the Putative Spirochaete Omp-like OMPP (Sp-Omp) Family (9.B.184) [96] have size, topological and sequence characteristics resembling those of *E*. *coli* OmpA and OmpW homologues, but the function of no member of this family has been established. This family will therefore not be considered further.

Cluster IV includes two families, the General Bacterial OMPP (GBP) Family (1.B.1) and the *Rhodobacter* PorCa OMPP (RPP) Family (1.B.7). The *Rhodobacter* OMPP was the first OMPP to have its high resolution structure solved [97]. Subsequently, the structures of several members of the GBP family were solved and all proved to consist of trimeric pores, each subunit having 16 β-TMSs, like the *Rhodobacter* OMPP [59]. That these two families belong to a single subcluster was therefore not unexpected.

Cluster V includes the well characterized FadL OMPP Family (1.B.9), concerned with transport of hydrophobic molecules such as fatty acids, benzene derivatives, hydrocarbons, hemin and salicylate esters [98] and the poorly characterized *Legionella* Major OMP (LM-OMP) Family (1.B.57) [99]. Members of these two families are of similar sizes (Table 1), and while FadL of *E*. *coli* is known to have 14 established β-TMSs, LM-OMP Family members are predicted to have 12–14 β-TMSs. Structural similarities with FadL seem likely.

Cluster VI consists of three OMPP families, the first well characterized Sugar OMPP (SP) Family (1.B.3) that includes the trimeric *E*. *coli* maltoOMPP with 18 established β-TMSs [100, 101] as well as the sucrose and β-glucoside OMPPs, and second, the much less well characterized Raffinose (RafY) Family (1.B.15), which includes the *E*. *coli* RafY OMPP that transports several oligosaccharides including the trisaccharide, raffinose [102, 103]. The OMPPs of these two families have overlapping specificities for oligosaccharides, and they are closer to each other on the tree than to any other OMPP family. However, a third family, the Chlamydial OMPP (CP) Family (1.B.2) also occurs in this cluster. The members of this family have similar sizes and topologies as the RafY family, and like maltoOMPP, these Chlamydial OMPPs, which are known to transport small nutrients, are homotrimers [104].

Branches (Clusters) VII, VIII and IX include just 1, 2 and 1 families, respectively. Moreover, the branch point for the two families in cluster VIII are so close to the center of the tree, it cannot be concluded with confidence that they are more closely related to each other than to the families in clusters VII and IX. These families are the uncharacterized Putative β-Barrel OMPP-4 (BBP4) Family (1.B.68) (branch VII), the *Porphyromonas gingivalis* OMPP (PorT) Family (1.B.44) (cluster VIII), the Outer Membrane Receptor (OMR) Family (1.B.14), (cluster VIII) and the Alginate Export OMPP (AEP) Family (1.B.13; cluster IX). Members of these families exhibit differing sizes and topologies with 8, 22, 8 and 18 predicted β-TMSs, respectively, in agreement with the fact that they stem from points near the center of the tree. Of these four families, only the OMR and AEP families include members that are functionally well characterized [105, 106].

Cluster X includes four families. The Cyclodextrin OMPP (CDP) Family (1.B.26) and the Oligogalacturonate OMPP (KdgM) Family (1.B.35) [107, 108], which includes the structurally characterized 12 TMS NanC OMPP [109], are families of polysaccharide export OMPPs that cluster closely together, a fact that is noteworthy since both are specific for complex carbohydrates. Members of these two families form β-barrels, but they differ in average protein size (about 350 aas versus 530 aas) and possibly topologies (14–16 predicted β-TMSs versus 12 established β-TMSs, respectively). Branching lower within cluster X is the OmpG OMPP (OmpG) Family (1.B.21), and closest to the center of the tree, we find the Fusobacterial Outer Membrane OMPP (FomA) Family (1.B.32). Proteins of these two families have about the same sizes and numbers of β-TMSs (14) as the CDP and KdgM families, but they are reported to catalyze non-specific export of small molecules, restricted only by the sizes of the substrates [110–112].

Cluster XI consists of just two families, the Nucleoside-specific Channel-forming Outer Membrane OMPP (Tsx) Family (1.B.10) [113] and the Capsule Biogenesis/Assembly (CBA) Family (1.B.73) [41]. They differ in protein sizes and numbers of β-TMSs (12 versus 18 established β-TMSs). The molecular functions of CBA family members are not well established, but Wzi of *E*. *coli*, a member of the CBA family, is a carbohydrate binding protein (lectin) that is somehow involved in extracellular capsule formation [114].

Cluster XII consists of two families, the *Campylobacter jejuni* Major Outer Membrane OMPP (MomP) Family (1.B.31) [88] and the Delta-Proteobacterial OMPP (Delta-OMPP) Family (1.B.61). The OMPPs of these two families are of similar sizes and putative topologies, but none is well characterized.

Cluster XIII includes three families. The poorly characterized Intimin/Invasin or Autotransporter-3 (AT-3) Family (1.B.54) and the Proteobacterial/Verrucomicrobial OMPP (PVP) Family (1.B.71) cluster more closely together than to the much better characterized, but more distantly related Mitochondrial and Plastid OMPP (MPP) Family (1.B.8) that includes outer mitochondrial membrane OMPPs called VDAC. While members of the first two of these families may consist of 12 β-TMS barrels, VDAC OMPPs have 19 established β-TMSs. The latter proteins can differ in cellular location, size and structure due to alternative splicing [115], but they always appear to form anion-selective pores.

Cluster XIV includes four families. The *Brucella-Rhizobium* OMPP (BRP) Family (1.B.4) consists of smallish proteins with about 260 aas and 12 established β-TMSs [116]. The closely related Protochlamydial Outer Membrane OMPP (PomS/T) Family (1.B.72) [68], also of 12 predicted β-TMSs, is known to transport anions selectively, but its physiological substrates are not known. The phylogenetically more distant *Coxiella* OMPP P1 (CPP1) Family (1.B.43) consists of OMPPs of a size similar to those of the BRP Family with 8–12 predicted β-TMSs. Finally the most distant member of cluster XIV, the OMC Family (1.B.70) [117] is so poorly characterized that an OMPP function is not established. These putative β-barrel OMPPs may have about 16 β-TMSs.

Cluster XV includes three families, all fairly distantly related, but two are more closely related to each other than they are to the third. The former two families are the Glucose-Selective OprB OMPP (OprB) Family (1.B.19) with protein sizes of about 460 aas and 16 established β-TMSs, and the Putative β-Barrel OMPP-2 (BBP2) Family (1.B.66) of unknown structure and function. It is presumed to function as an OMPP, based solely on the phylogenetic analyses reported here. The last and more distantly related family in cluster XV, is the large and well characterized Opr Family (1.B.25). These proteins are of about 430 aas with 18 established β-TMSs. They transport a variety of small molecules including amino acids, peptides, phenolic compounds, antibiotics and sugar derivatives [118, 119]. It can be concluded that like many other OMPPs, these channel proteins exhibit broad specificities and are simply size limited.

### Protein phylogenetic trees for Superfamilies II and III

**Fig 6:**
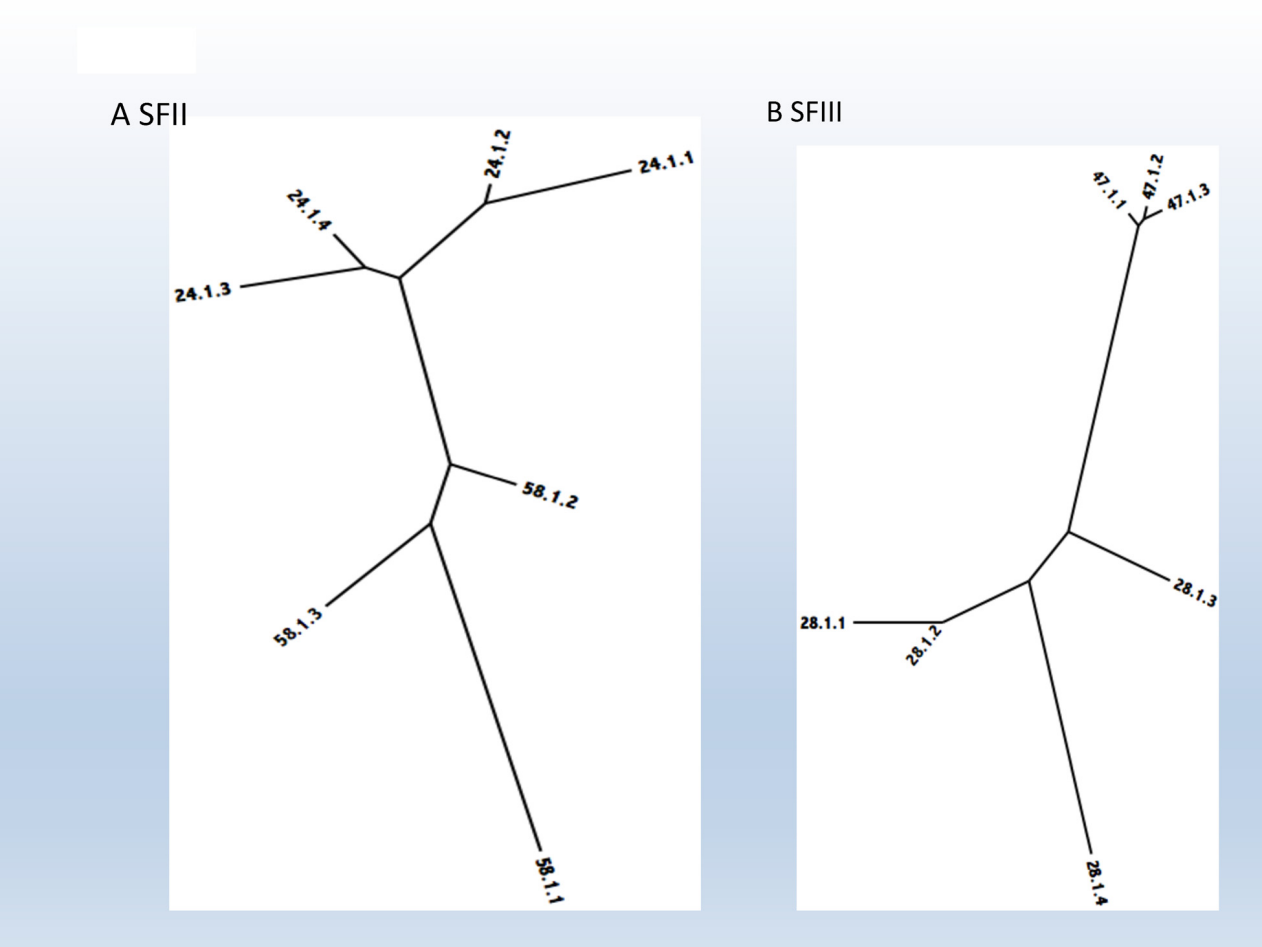
Phylogenetic trees based on the SuperfamilyTree I program for Superfamily II (A) and Superfamily III (B). The TC numbers of the proteins in TC subclass 1.B are provided.

Acid fast Gram-positive Actinobacteria have OMPPs of two types, β-barrel and α-helical, in their outer membranes (see Table 1 and TCDB [120, 121]). Two of these OMPP families, the Mycobacterial OMPP (MBP or MspA) Family (1.B.24) and the Nocardial Heterooligomeric Cell Wall Channel (NfpA/B) Family (1.B.58) are believed to consist of β-barrels and comprise Superfamily II [122, 123]. These two families include proteins, most of which are of 200–290 aas with a single N-terminal α-helical TMS followed by a proposed β-barrel OMPP-type structure. The tree shown in Fig 6A reveals that, as expected, members of these two families segregate into two distinct clusters. Because the proteins in SFII are quite similar, it is not surprising that the SFT1 and ClustalX/FigTree trees were in good agreement (compare Fig 6A and S2A Fig).

Superfamily III (Fig 6B) includes the Plastid Outer Envelope OMPP of 24 KDa (OEP24; 1.B.28) and the Plastid Outer Envelope OMPP of 37 KDa (OEP37; 1.B.47) Families. These β-barrel OMPPs are of about 220 and 330 aas in size, respectively, and are predicted to have 12 and 14 β-TMSs, respectively. Presumably, because of their simplicities, the SFT1 tree proved to be in good agreement with the tree based on the ClustalX multiple alignment (see Fig 6B and S2B Fig).

### OMPP repeat sequences

**Fig 7:**
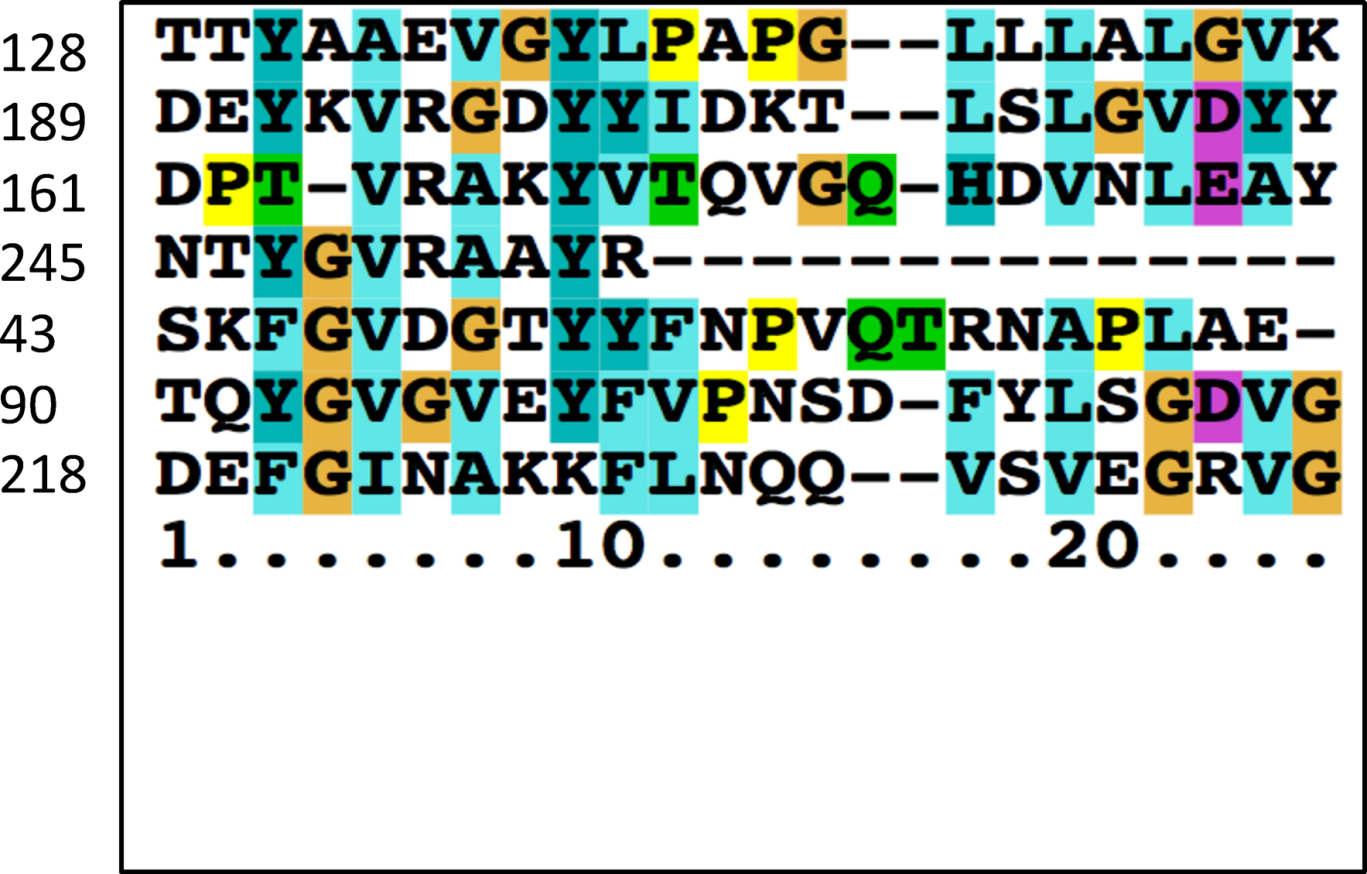
Putative β-hairpin repeats in the β-barrel OMPP, 1.B.1.2.1. Repeats were identified using the HHRepID program and aligned using the MEME program. Residue position is indicated on the left. Positions with conservation are shaded as dictated by the program.

Attempts were made to identify the proposed 8 β-TMS repeats in the 16 β-TMS proteins of Superfamilies I & II using AncientRep [36], but these attempts were unsuccessful. We then used the HHrepID program to look for internal repeats and the PRED-TMBB program to predict the β-TMSs. While these programs also failed to identify the proposed eight β-TMS repeats, they did identify what appeared to be hairpin repeats with P values between e^-2^ and e^-10^. For example, 1.B.1.1.1 and 1.B.1.1.2, both of which consist of proteins with 16 β-TMSs, appeared to have four tandem 2 TMS β-hairpin repeats corresponding to predicted β-TMSs 8 & 9, 10 & 11, 12 & 13, and 14 & 15 with P values between e^-4^ and e^-7^. On the other hand, 1.B.1.2.1, which was predicted to have 14 β-TMSs, appeared to have at least five and possibly seven β-hairpin repeats, starting with β-TMS 4 and ending with β-TMS 13, with P values between e^-3^ and e^-9^ (Fig 7). Different proteins in family 1.B.4 were predicted to have variable numbers of β-TMSs and anywhere between 1 and 3 putative β-hairpin repeats. Several proteins in family 1.B.6 were predicted to have at least three β-hairpin repeats with P values in the same range. Putative adjacent β-hairpin repeats were also identified in families 9 (up to 7 repeats), 14 (up to 8 repeats), and 24 (up to 5 repeats). However, hairpin repeats were not identified in members of several families including 28, 47 and 58. Interestingly, four adjacent putative 1 β-TMS repeats were identified in one member of family 28, the protein with TC# 1.B.28.1.4. It is worth noting that families 28 and 47, where no hairpin repeats were identified, belong to Superfamily III, and families 24 and 58, which comprise Superfamily II, in general, did not exhibit identifiable repeats. However, in one protein within family 24, 1.B.24.1.4, five β-hairpin repeats were predicted. Because of concerns about convergent sequence evolution and the short lengths of these sequences, we do not consider it certain that these results can be interpreted in terms of a primordial hairpin structure being the precursor of the proteins in Superfamilies I & II although a previous report came to this conclusion [31].

## Discussion

In 1997, Paulsen et al., published a review concerning a family of Gram-negative outer membrane factors involved in the export of proteins, complex carbohydrates, drugs and heavy metals [124]. They showed that these substrates could be exported via a complex of three proteins, an inner membrane transporter of any one of several types that provided the energy for export across the entire cell envelope, a “Membrane Fusion Protein” (MFP) that may serve primarily as an “adaptor”, joining the two membranes of the Gram-negative bacterial envelope, and the above mentioned Outer Membrane Factor that provided the β-barrel pore through which the substrates permeate the outer membrane. Since then, sixteen mechanisms of protein export across one or the other of the two membranes of the Gram-negative bacterial envelope or both have been described, and all require the presence of a pore-forming outer membrane protein (OMPP) [125, 126]. However, outer membrane pore-forming proteins can function in non-specific or substrate-specific transport into or out of the cell, and many of these are not coupled to an energy input source. They can therefore facilitate transport in both directions [5, 127]. Few attempts have been made to classify these proteins into families and superfamilies. As part of a massive attempt to categorize all well conserved cellular proteins, the Pfam database has used Hidden Markov Models (HMMs) and UniProtKB reference proteins to categorize outer membrane pore-forming proteins among others [128]. The current release of this database (Pfam 29.0) includes 16,295 entries classified into 559 Clans, and relationships between families within a clan (i.e., a superfamily) have been suggested using newly prepared bioinformatic tools [128].

Based in part on Pfam, two other databases, devoted exclusively to β-barrel outer membrane proteins from Gram-negative bacteria, have been derived [129]. They define families including about 20,000 and 80,000 proteins respectively, again based largely on HMMs but also using the transitivity rule and 3-d structures when available [130]. The Pfam family/clans are presented in Table 1, last column, and comparisons between TCDB and Pfam are discussed in the text as well as in reference 131. It is apparent that many of the families in TCDB often correlate with those in Pfam and OMPdb, and some of the TC families found within a single superfamily are also found in single clans in Pfam.

While Pfam, HHomp and OMPdb are based substantially on HMMs as noted above, TCDB is based largely on the Superfamily Principle, also known as the Transitivity Rule. Surprisingly, perhaps, there is considerable concordance. We have screened OMPdb and Pfam for overlap, insuring that all of the members in these databases are included in TCDB (see reference [131].

The first comprehensive analysis of outer membrane β-barrel proteins was that of Remmer et al., 2010 [37]. These investigators used three criteria for suggesting homology as discussed in the Introduction. Despite the differences in topology (8–24 β-strands in the barrel) they provided evidence that many β-barrel porins “arose by amplification and recombination of short peptide modules”. They did not, however, assign families to superfamilies or attempt phylogenetic analyses of the proteins within these superfamilies. Using defined criteria for quantitative homology assignment and the Superfamily Tree programs, SFT1 and 2, to construct phylogenetic trees, we were able to correct for these deficiencies as reported here.

We have identified 76 TC families of outer membrane OMPPs, most being from Gram-negative bacteria, but some being from outer membrane-possessing Gram-positive Firmicutes and Actinobacteria, and some being from eukaryotic organelles. Of the Gram-negative bacterial OMPPs, 47 of the families appear to form a single large superfamily which we have designated SFI. Phylogenetic analyses using SFT1 and SFT2 [11, 12, 21, 35, 42] revealed for the first time that members of each of these families cluster coherently together in almost all cases, and that the families fall into fifteen clusters. Because these proteins are highly divergent in sequence, it is not surprising that ClustalX/FigTree phylogenetic trees were not reliable as documented previously for other sequence-divergent superfamilies [11, 12, 21, 35, 42]. The SFT2 tree, showing family relationships (Fig 5), in general, confirmed the SFT1 tree of the proteins (not shown) with a few minor exceptions that proved in every case to be uncertainties due to deep branching. The analyses revealed common functions and/or topologies among some but not all of the most closely related families. However, even within a single family, or within a closely related set of families, functions and topologies may differ substantially. These facts must reflect the ease with which OMPPs change their numbers of β-strands and alter their pore sizes as well as their substrate specificities during their evolutionary divergence. In this respect it is interesting that Korkmaz et al [132] duplicated the last 38aa β-hairpin at the end of the 14 β-stranded OmpG of *E*. *coli* (TC #1.B.21.1.) to produce a 16 β-TMS OMPP with similar properties and stabilities, but altered pH sensitivity. Thus, duplication of a β-hairpin structure has been documented with retention of a functional β-barrel.

We noted that OMPPs from Gram-negative bacteria are often related to each other and reside in SFI, that the small SFII includes β-barrel proteins only from Actinobacteria, and that the SFIII proteins derive exclusively from chloroplasts of eukaryotes. Three families of outer membrane proteins from Actinobacteria and two families from eukaryotes are known to consist of transmembrane α-helices [65, 133]. Two of the former and two of the latter comprise SFIV and SFV, respectively. Of the seventy one putative β-barrel OMPP families, many of the families in each superfamily may have related topologies (8 and 16 β-TMSs for Superfamilies I & II, but 12 and 14 β-TMSs for Superfamily III). Because the sequences that comprise SFII, and those that comprise SFIII, are similar within each superfamily, it was not surprising that the SFT trees showed good agreement with the trees based on multiple alignments (compare Fig 6 with S2 Fig). For SFI, the demonstration of homology and construction of phylogenetic trees provided the first evidence that several families of structurally and functionally uncharacterized proteins are in fact, OMPPs. Since these proteins are extremely divergent in sequence, it is not surprising that trees based on multiple alignments proved inaccurate. Moreover, some families in SF1 have proteins from different phyla. Sequence divergence between phyla has contributed substantially to the tremendous diversity of SFI. These results should serve as guides for further molecular biological experimentation.

The proteins that reside in β-barrel OMPP families not included in one of the identified β-TMS superfamilies in general proved to have a very different distribution of predicted topologies from those of Superfamilies I-III (Fig 3). This observation provides preliminary evidence that many of these outlying families may not be related to SFs I-III, and may instead, have evolved independently. Thus, the dominant topology for these families appears to involve twelve β-strand barrels (8 of 21 families), and no family of these 21 families exhibited the 16 or 18 β-TMS protein topology found in SFI as two of the most common topological types. This suggestion is in agreement with our inability to demonstrate homology between members of the five recognized OMPP superfamilies classified here. However, we were not able to document this proposal, for example, by showing that the route of evolution taken for the appearance of any of these proteins differs from those taken by other OMPPs. Such studies remain work for future investigations.

We noted that major topologies in SFs I and II involve 16 and 8 β-TMS topologies. This observation suggested the possibility that the larger of these proteins arose by intragenic duplication events, where the precursors were the smaller of these homologous proteins. Our attempts to demonstrate this pathway were not successful, although Arnold et al provided evidence that artificial internal duplication of 8 β-TMS OMPPs can give rise to 16 β-TMS OMPPs that are fully functional [62]. We could, however, observe limited similarities between adjacent 2 TMS hairpin structures in a number of these families in agreement with the observations of Remmert et al. [31]. The demonstration of apparently homologous adjacent hairpin structures in β-barrel proteins suggests that topological variations among these proteins could have arisen by gain or loss of β-hairpin structures. However, this does not eliminate the possibility of larger scale duplications such as the proposed 8 β-strand duplications to give 16 β-strands as proposed by Arnold et al. [62]. This last possibility is more in agreement with observations of intragenic duplication as occurred in most α-type integral membrane transport proteins [7]. Nevertheless, it should be kept in mind that these two transporter types (α and β) may have evolved via very different routes.

Of the 76 families of OMPPs described here, we could identify phylogenetic relationships for many of these families, revealing which families most recently diverged from common ancestors. These proteins were additionally analyzed for topology with the observation that at least in the large Superfamily I, the gain and/or loss of β-strands or of β-hairpin structures may have occurred repeatedly during the evolutionary divergence of members of this superfamily. We do, however, suggest that not all β-barrel OMPPs derived from a common β-hairpin structure. Interestingly, we have identified naturally occurring proteins in which two or even three OMPP domains are present within a single polypeptide chain. These may function to coordinate the transport of physiologically related compounds. Further studies will be required to define the specific pathways that gave rise to these proteins of dissimilar topologies.

After completing the work described in this article, evidence for five new (putative) OMPP families has appeared. (1) The Oep23 Family (1.B.77) includes a characterized OMPP in the outer envelope of chloroplasts [134]. Bacterial homologues, particularly from Actinobacteria, have been identified. We have not been able to show that this family is related to any other OMPP family. (2) The Electron Transport-associated OMPP (ETOMPP) Family (1.B.78) includes proteobacterial members involved in the transenvelope transport of electrons, allowing extracellular metal oxidoreduction [135, 136]. This family proved, by our criteria, to be a member of SFI. (3) The SpmT Family (1.B.79), with members in Actinobacteria, is a putative OMPP (N-terminus) sphingomyelinase (C-terminus) fusion protein. Evidence that the OMPP domain can transport glucose and phosphocholine has been presented [137]. (4) The Putative Trans-Outer Membrane Electron Flow OMPP (TOM-EF) Family (1.B.80) appears to be very distantly related to members of the ETOMPP Family (TC#1.B.78). It probably serves the same or a closely related function [138]. Both families function with periplasmic and outer membrane cytochrome c proteins and may belong to SFI. (5) Finally, the most recently identified putative porin family (DUF2490; TC#1.B.81) may also belong to SFI, but its functions are not established.

In subclass 9.B of TCDB, we have listed 14 additional families, which, on the basis of tentative predictions, may consist of OMPP proteins, even though in no case has an OMPP function been established, and in no case has homology with members of an established OMPP family been demonstrated. These putative OMPP families in TC subclass 9.B include families 138, 153, 155, 161–165, 167, 168, 170–172 and 184. Clearly, many novel OMPP families are in need of functional and structural characterization.

## Supporting Information

S1 Fig Clustal X tree for representative Superfamily I proteins. (PDF)

S2 Fig Clustal X trees for representative protein members of Superfamily II (A) and Superfamily III (B).
(PDF)
