# Supplementary material for: Properties and Phylogeny of 76 Families of Bacterial and Eukaryotic Organellar Outer Membrane Pore-Forming Proteins
Source: PLoS One. 2016 Apr 11;11(4):e0152733. doi: 10.1371/journal.pone.0152733 (PMC4827864; doi:10.1371/journal.pone.0152733)
Supplement: S1 Fig — (PDF) [file pone.0152733.s001.pdf]

Fig. S1. PORIN SUPERFAMILY I: Clustal X

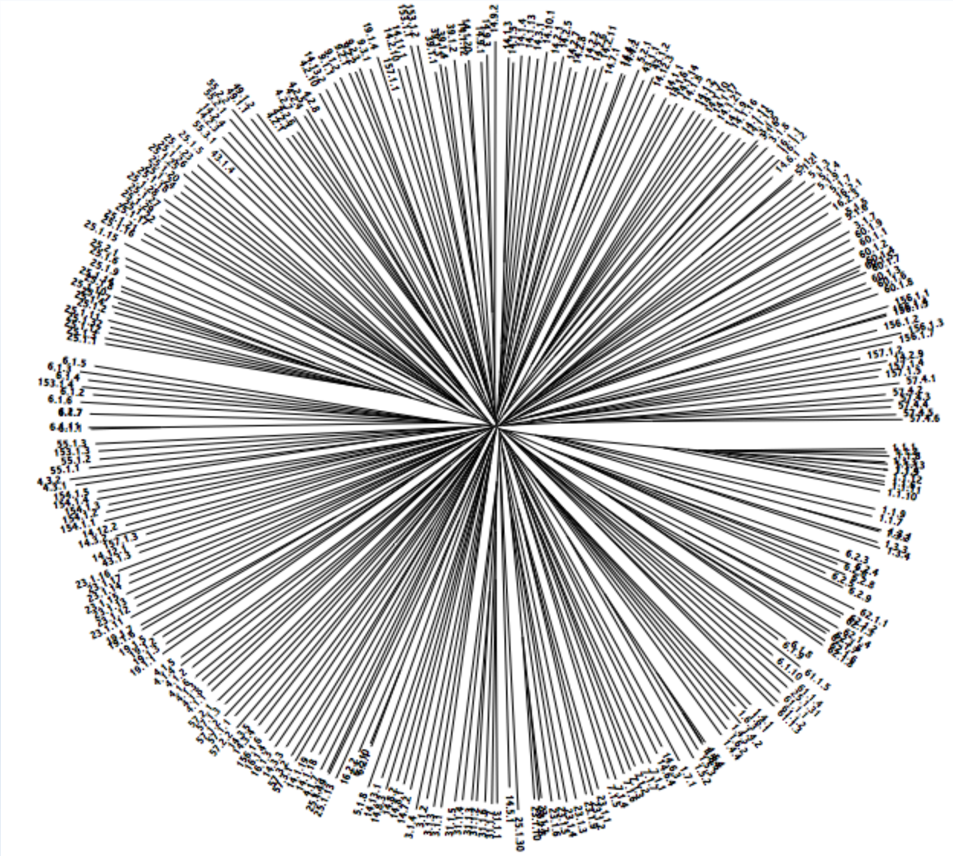

Supplementary Figure S1: Clustal X tree for representative Superfamily I proteins.
