# Supplementary material for: Properties and Phylogeny of 76 Families of Bacterial and Eukaryotic Organellar Outer Membrane Pore-Forming Proteins
Source: PLoS One. 2016 Apr 11;11(4):e0152733. doi: 10.1371/journal.pone.0152733 (PMC4827864; doi:10.1371/journal.pone.0152733)
Supplement: S2 Fig — Clustal X trees for representative protein members of Superfamily II (A) and Superfamily III (B). (PDF) [file pone.0152733.s002.pdf]

Fig.S2(a). PORIN SUPERFAMILY II: Clustal X

Fig.S2(b). PORIN SUPERFAMILY III: Clustal X

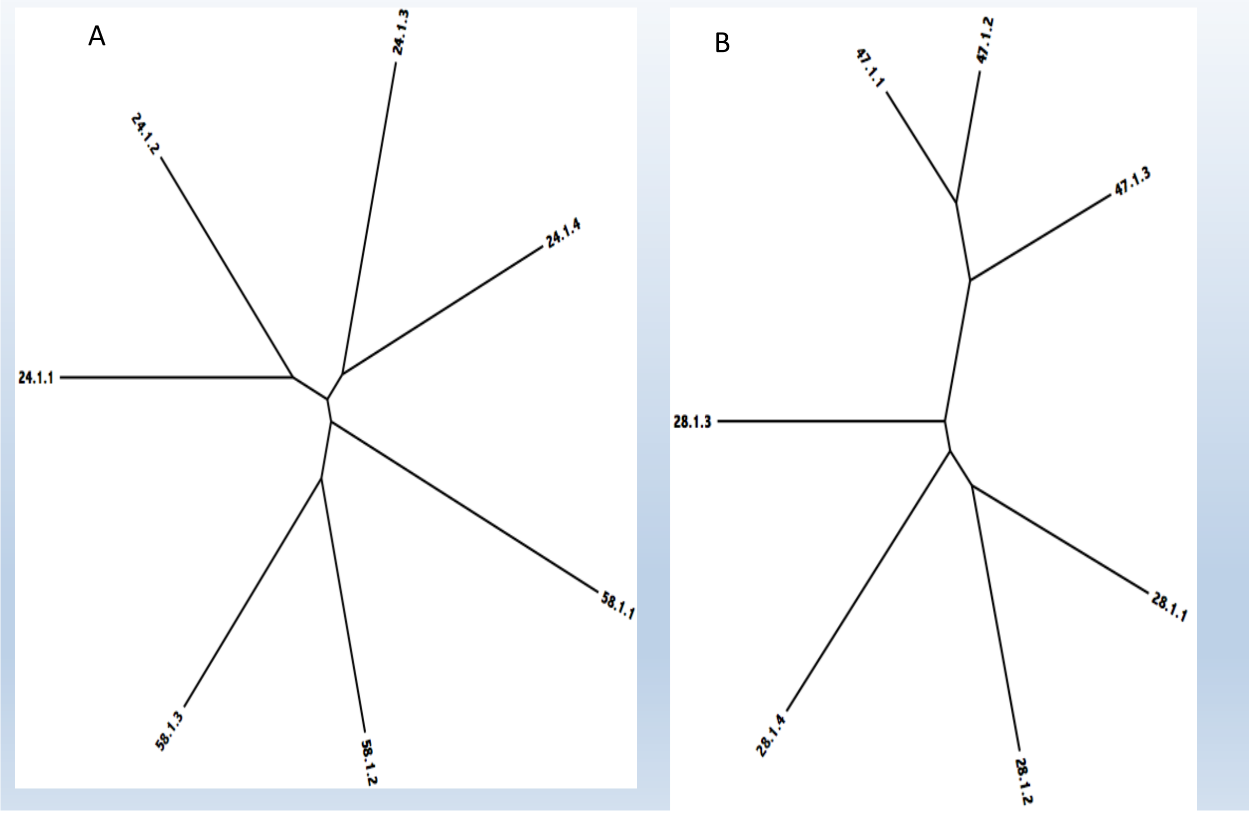

Supplementary Figures S2: Clustal X trees for representative protein members of Superfamily II (a) and Superfamily III (b).
